# External validation of prognostic models predicting pre-eclampsia: individual participant data meta-analysis

**DOI:** 10.1186/s12916-020-01766-9

**Published:** 2020-11-02

**Authors:** Kym I. E. Snell, John Allotey, Melanie Smuk, Richard Hooper, Claire Chan, Asif Ahmed, Lucy C. Chappell, Peter Von Dadelszen, Marcus Green, Louise Kenny, Asma Khalil, Khalid S. Khan, Ben W. Mol, Jenny Myers, Lucilla Poston, Basky Thilaganathan, Anne C. Staff, Gordon C. S. Smith, Wessel Ganzevoort, Hannele Laivuori, Anthony O. Odibo, Javier Arenas Ramírez, John Kingdom, George Daskalakis, Diane Farrar, Ahmet A. Baschat, Paul T. Seed, Federico Prefumo, Fabricio da Silva Costa, Henk Groen, Francois Audibert, Jacques Masse, Ragnhild B. Skråstad, Kjell Å. Salvesen, Camilla Haavaldsen, Chie Nagata, Alice R. Rumbold, Seppo Heinonen, Lisa M. Askie, Luc J. M. Smits, Christina A. Vinter, Per Magnus, Kajantie Eero, Pia M. Villa, Anne K. Jenum, Louise B. Andersen, Jane E. Norman, Akihide Ohkuchi, Anne Eskild, Sohinee Bhattacharya, Fionnuala M. McAuliffe, Alberto Galindo, Ignacio Herraiz, Lionel Carbillon, Kerstin Klipstein-Grobusch, Seon Ae Yeo, Joyce L. Browne, Karel G. M. Moons, Richard D. Riley, Shakila Thangaratinam, for the IPPIC Collaborative Network, Alex Kwong, Ary I. Savitri, Kjell Å. Salvesen, Sohinee Bhattacharya, Cuno S. P. M. Uiterwaal, Annetine C. Staff, Louise B. Andersen, Elisa L. Olive, Christopher Redman, George Daskalakis, Maureen Macleod, Baskaran Thilaganathan, Javier Arenas Ramírez, Jacques Massé, Asma Khalil, Francois Audibert, Per M. Magnus, Anne K. Jenum, Ahmet Baschat, Akihide Ohkuchi, Fionnuala M. McAuliffe, Jane West, Lisa M. Askie, Fionnuala Mone, Diane Farrar, Peter A. Zimmerman, Luc J. M. Smits, Catherine Riddell, John C. Kingdom, Joris van de Post, Sebastián E. Illanes, Claudia Holzman, Sander M. J. van Kuijk, Lionel Carbillon, Pia M. Villa, Anne Eskild, Lucy Chappell, Federico Prefumo, Luxmi Velauthar, Paul Seed, Miriam van Oostwaard, Stefan Verlohren, Lucilla Poston, Enrico Ferrazzi, Christina A. Vinter, Chie Nagata, Mark Brown, Karlijn C. Vollebregt, Satoru Takeda, Josje Langenveld, Mariana Widmer, Shigeru Saito, Camilla Haavaldsen, Guillermo Carroli, Jørn Olsen, Hans Wolf, Nelly Zavaleta, Inge Eisensee, Patrizia Vergani, Pisake Lumbiganon, Maria Makrides, Fabio Facchinetti, Evan Sequeira, Robert Gibson, Sergio Ferrazzani, Tiziana Frusca, Jane E. Norman, Ernesto A. Figueiró-Filho, Olav Lapaire, Hannele Laivuori, Jacob A. Lykke, Agustin Conde-Agudelo, Alberto Galindo, Alfred Mbah, Ana P. Betran, Ignacio Herraiz, Lill Trogstad, Gordon G. S. Smith, Eric A. P. Steegers, Read Salim, Tianhua Huang, Annemarijne Adank, Jun Zhang, Wendy S. Meschino, Joyce L. Browne, Rebecca E. Allen, Fabricio da Silva Costa, Kerstin Klipstein-Grobusch, Caroline A. Crowther, Jan S. Jørgensen, Jean-Claude Forest, Alice R. Rumbold, Ben W. Mol, Yves Giguère, Louise C. Kenny, Wessel Ganzevoort, Anthony O. Odibo, Jenny Myers, SeonAe Yeo, Francois Goffinet, Lesley McCowan, Eva Pajkrt, Bassam G. Haddad, Gustaaf Dekker, Emily C. Kleinrouweler, Édouard LeCarpentier, Claire T. Roberts, Henk Groen, Ragnhild B. Skråstad, Seppo Heinonen, Kajantie Eero

**Affiliations:** 1 Centre for Prognosis Research, School of Primary, Community and Social Care, Keele University, Keele, UK; 2 Barts Research Centre for Women’s Health (BARC), Barts and the London School of Medicine and Dentistry, Queen Mary University of London, London, UK; 3 Pragmatic Clinical Trials Unit, Barts and the London School of Medicine and Dentistry, Queen Mary University of London, London, UK; 4 MirZyme Therapeutics, Innovation Birmingham Campus, Birmingham, UK; 5 Department of Women and Children’s Health, School of Life Course Sciences, King’s College London, London, UK; 6 Action on Pre-eclampsia (APEC) Charity, Worcestershire, UK; 7 Faculty Health & Life Sciences, University of Liverpool, Liverpool, UK; 8 Fetal Medicine Unit, St George’s University Hospitals NHS Foundation Trust and Molecular and Clinical Sciences Research Institute, St George’s University of London, London, UK; 9 Department of Obstetrics and Gynaecology, Monash University, Monash Medical Centre, Clayton, Victoria Australia; 10 Maternal and Fetal Health Research Centre, Manchester Academic Health Science Centre, University of Manchester, Central Manchester NHS Trust, Manchester, UK; 11 Division of Obstetrics and Gynaecology, Oslo University Hospital, and Faculty of Medicine, University of Oslo, Oslo, Norway; 12 Department of Obstetrics and Gynaecology, NIHR Biomedical Research Centre, Cambridge University, Cambridge, UK; 13 Department of Obstetrics, Amsterdam UMC University of Amsterdam, Amsterdam, The Netherlands; 14 Department of Medical and Clinical Genetics, University of Helsinki and Helsinki University Hospital, Helsinki, Finland; 15 Institute for Molecular Medicine Finland, Helsinki Institute of Life Science, University of Helsinki, Helsinki, Finland; 16 Department of Obstetrics and Gynecology, Faculty of Medicine and Health Technology, Tampere University Hospital and Tampere University, Tampere, Finland; 17 University of South Florida, Tampa, FL USA; 18 Department of Obstetrics and Gynaecology, University Hospital de Cabueñes, Gijón, Spain; 19 Maternal-Fetal Medicine Division, Department OBGYN, Mount Sinai Hospital, University of Toronto, Toronto, Canada; 20 Department of Obstetrics and Gynecology, National and Kapodistrian University of Athens, Alexandra Hospital, Athens, Greece; 21 Bradford Institute for Health Research, Bradford Teaching Hospitals, Bradford, UK; 22 Johns Hopkins Center for Fetal Therapy, Department of Gynecology & Obstetrics, Johns Hopkins University School of Medicine, Baltimore, MD USA; 23 Department of Obstetrics and Gynaecology, University of Brescia, Brescia, Italy; 24 Department of Gynecology and Obstetrics, Ribeirão Preto Medical School, University of São Paulo, Ribeirão Preto, São Paulo, Brazil; 25 Department of Epidemiology, University of Groningen, University Medical Center Groningen, Groningen, The Netherlands; 26 Department of Obstetrics and Gynecology, CHU Ste Justine, Université de Montréal, Montreal, Canada; 27 Department of Molecular Biology, Medical Biochemistry and Pathology, Laval University, Quebec City, Canada; 28 Department of Clinical and Molecular Medicine, Faculty of Medicine and Health Sciences, Norwegian University of Science and Technology – NTNU, Trondheim, Norway; 29 Department of Clinical Pharmacology, St. Olav University Hospital, Trondheim, Norway; 30 Department of Obstetrics and Gynecology, Trondheim University Hospital, Trondheim, Norway; 31 Department of Laboratory Medicine, Children’s and Women’s Health, Norwegian University of Science and Technology, Trondheim, Norway; 32 Department of Obstetrics and Gynaecology, Akershus University Hospital, Lørenskog, Norway; 33 Department of Education for Clinical Research, National Center for Child Health and Development, Tokyo, Japan; 34 South Australian Health and Medical Research Institute and Robinson Research Institute, The University of Adelaide, Adelaide, Australia; 35 Department of Obstetrics and Gynaecology, University of Helsinki and Helsinki University Hospital, Helsinki, Finland; 36 NHMRC Clinical Trials Centre, University of Sydney, Sydney, Australia; 37 Care and Public Health Research Institute, Maastricht University Medical Centre, Maastricht, The Netherlands; 38 Department of Gynecology and Obstetrics, Odense University Hospital, University of Southern Denmark, Odense, Denmark; 39 Centre for Fertility and Health, Norwegian Institute of Public Health, Oslo, Norway; 40 National Institute for Health and Welfare, Helsinki, Finland; 41 Children’s Hospital, University of Helsinki and Helsinki University Hospital, Helsinki, Finland; 42 General Practice Research Unit (AFE), Department of General Practice, Institute of Health and Society, Faculty of Medicine, University of Oslo, Oslo, Norway; 43 Institute for Clinical Research, University of Southern Denmark, Odense, Denmark; 44 Department of Obstetrics and Gynecology, Odense University Hospital, Odense, Denmark; 45 MRC Centre for Reproductive Health, University of Edinburgh, Edinburgh, UK; 46 Department of Obstetrics and Gynecology, Jichi Medical University School of Medicine, Shimotsuke-shi, Tochigi Japan; 47 Institute of Clinical Medicine, University of Oslo, Oslo, Norway; 48 Obstetrics & Gynaecology, Institute of Applied Health Sciences, School of Medicine, Medical Sciences and Nutrition, University of Aberdeen, Aberdeen, UK; 49 UCD Perinatal Research Centre, School of Medicine, University College Dublin, National Maternity Hospital, Dublin, Ireland; 50 Fetal Medicine Unit, Maternal and Child Health and Development Network (SAMID), Department of Obstetrics and Gynaecology, Hospital Universitario, Instituto de Investigación Hospital, Universidad Complutense de Madrid, Madrid, Spain; 51 Department of Obstetrics and Gynecology, Assistance Publique-Hôpitaux de Paris Université Paris, Paris, France; 52 Julius Centre for Health Sciences and Primary Care, University Medical Centre Utrecht, Utrecht University, Utrecht, The Netherlands; 53 University of North Carolina at Chapel Hill, Chapel Hill, NC USA; 54 Cochrane Netherlands, Utrecht, The Netherlands; 55 Institute of Metabolism and Systems Research, WHO Collaborating Centre for Women’s Health, University of Birmingham, Birmingham, UK

**Keywords:** Pre-eclampsia, External validation, Prediction model, Individual participant data

## Abstract

**Background:**

Pre-eclampsia is a leading cause of maternal and perinatal mortality and morbidity. Early identification of women at risk during pregnancy is required to plan management. Although there are many published prediction models for pre-eclampsia, few have been validated in external data. Our objective was to externally validate published prediction models for pre-eclampsia using individual participant data (IPD) from UK studies, to evaluate whether any of the models can accurately predict the condition when used within the UK healthcare setting.

**Methods:**

IPD from 11 UK cohort studies (217,415 pregnant women) within the International Prediction of Pregnancy Complications (IPPIC) pre-eclampsia network contributed to external validation of published prediction models, identified by systematic review. Cohorts that measured all predictor variables in at least one of the identified models and reported pre-eclampsia as an outcome were included for validation. We reported the model predictive performance as discrimination (*C*-statistic), calibration (calibration plots, calibration slope, calibration-in-the-large), and net benefit. Performance measures were estimated separately in each available study and then, where possible, combined across studies in a random-effects meta-analysis.

**Results:**

Of 131 published models, 67 provided the full model equation and 24 could be validated in 11 UK cohorts. Most of the models showed modest discrimination with summary *C*-statistics between 0.6 and 0.7. The calibration of the predicted compared to observed risk was generally poor for most models with observed calibration slopes less than 1, indicating that predictions were generally too extreme, although confidence intervals were wide. There was large between-study heterogeneity in each model’s calibration-in-the-large, suggesting poor calibration of the predicted overall risk across populations. In a subset of models, the net benefit of using the models to inform clinical decisions appeared small and limited to probability thresholds between 5 and 7%.

**Conclusions:**

The evaluated models had modest predictive performance, with key limitations such as poor calibration (likely due to overfitting in the original development datasets), substantial heterogeneity, and small net benefit across settings. The evidence to support the use of these prediction models for pre-eclampsia in clinical decision-making is limited. Any models that we could not validate should be examined in terms of their predictive performance, net benefit, and heterogeneity across multiple UK settings before consideration for use in practice.

**Trial registration:**

PROSPERO ID: CRD42015029349.

## Background

Pre-eclampsia, a pregnancy-specific condition with hypertension and multi-organ dysfunction, is a leading contributor to maternal and offspring mortality and morbidity. Early identification of women at risk of pre-eclampsia is key to planning effective antenatal care, including closer monitoring or commencement of prophylactic aspirin in early pregnancy to reduce the risk of developing pre-eclampsia and associated adverse outcomes. Accurate prediction of pre-eclampsia continues to be a clinical and research priority [1, 2]. To-date, over 120 systematic reviews have been published on the accuracy of various tests to predict pre-eclampsia; more than 100 prediction models have been developed using various combinations of clinical, biochemical, and ultrasound predictors [3–6]. However, no single prediction model is recommended by guidelines to predict pre-eclampsia. Risk stratification continues to be based on the presence or absence of individual clinical markers, and not by multivariable risk prediction models.

Any recommendation to use a prediction model in clinical practice must be underpinned by robust evidence on the reproducibility of the models, their predictive performance across various settings, and their clinical utility. An individual participant data (IPD) meta-analysis that combines multiple datasets has great potential to externally validate existing models [7–10]. In addition to increasing the sample size beyond what is feasibly achievable in a single study, access to IPD from multiple studies offers the unique opportunity to evaluate the generalisability of the predictive performance of existing models across a range of clinical settings. This approach is particularly advantageous for predicting the rare but serious condition of early-onset pre-eclampsia that affects 0.5% of all pregnancies [11].

We undertook an IPD meta-analysis to externally validate the predictive performance of existing multivariable models to predict the risk of pre-eclampsia in pregnant women managed within the National Health Service (NHS) in the UK and assessed the clinical utility of the models using decision curve analysis.

## Methods

### International Prediction of Pregnancy Complications (IPPIC) Network

We undertook a systematic review of reviews by searching Medline, Embase, and the Cochrane Library including DARE (Database of Abstracts of Reviews of Effects) databases, from database inception to March 2017, to identify relevant systematic reviews on clinical characteristics, biochemical, and ultrasound markers for predicting pre-eclampsia [12]. We then identified research groups that had undertaken studies reported in the systematic reviews and invited the authors of relevant studies and cohorts with data on prediction of pre-eclampsia to share their IPD [13] and join the IPPIC (International Prediction of Pregnancy Complications) Collaborative Network. We also searched major databases and data repositories, and directly contacted researchers to identify relevant studies, or datasets that may have been missed, including unpublished research and birth cohorts. The Network includes 125 collaborators from 25 countries, is supported by the World Health Organization, and has over 5 million IPD containing information on various maternal and offspring complications. Details of the search strategy are given elsewhere [12].

### Selection of prediction models for external validation

We updated our previous literature search of prediction models for pre-eclampsia [3] (July 2012–December 2017), by searching Medline via PubMed. Details of the search strategy and study selection are given elsewhere (Supplementary Table S1, Additional file 1) [12]. We evaluated all prediction models with clinical, biochemical, and ultrasound predictors at various gestational ages (Supplementary Table S2, Additional file 1) for predicting any, early (delivery < 34 weeks), and late (delivery ≥ 34 weeks’ gestation) onset pre-eclampsia. We did not validate prediction models if they did not provide the full model equation (including the intercept and predictor effects), if any predictor in the model was not measured in the validation cohorts, or if the outcomes predicted by the model were not relevant.

### Inclusion criteria for IPPIC validation cohorts

We externally validated the models in IPPIC IPD cohorts that contained participants from the UK (IPPIC-UK subset) to determine their performance within the context of the UK healthcare system and to reduce the heterogeneity in the outcome definitions [14, 15]. We included UK participant whole datasets and UK participant subsets of international datasets (where country was recorded). If a dataset contained IPD from multiple studies, we checked the identity of each study to avoid duplication. We excluded cohorts if one or more of the predictors (i.e. those variables included in the model’s equation) were not measured or if there was no variation in the values of model predictors across individuals (i.e. every individual had the same predicted probability due to strict eligibility criteria in the studies). We also excluded cohorts where no individuals or only one individual developed pre-eclampsia. Since the published models were intended to predict the risk of pre-eclampsia in women with singleton pregnancies only, we excluded women with multi-foetal pregnancies.

### IPD collection and harmonisation

We obtained data from cohorts in prospective and retrospective observational studies (including cohorts nested within randomised trials, birth cohorts, and registry-based cohorts). Collaborators sent their pseudo-anonymised IPD in the most convenient format for them, and we then formatted, harmonised, and cleaned the data. Full details on the eligibility criteria, selection of the studies and datasets, and data preparation have previously been reported in our published protocol [13].

### Quality assessment of the datasets

Two independent reviewers assessed the quality of each IPD cohort using a modified version of the PROBAST (Prediction study Risk of Bias Assessment) tool [16]. The tool assesses the quality of the cohort datasets and individual studies, and we used three of the four domains: participant selection, predictors, and outcomes. The fourth domain ‘analysis’ was not relevant for assessing the quality of the collected data, as we performed the prediction model analyses ourselves since we had access to the IPD. We classified the risk of bias to be low, high, or unclear for each of the relevant domains. Each domain included signalling questions that are rated as ‘yes’, ‘probably yes’, ‘probably no’, ‘no’, or ‘no information’. Any signalling question that was rated as ‘probably no’ or ‘no’ was considered to have potential for bias and was classed as high risk of bias in that domain. The overall risk of bias of an IPD dataset was considered to be low if it scored low in all domains, high if any one domain had a high risk of bias, and unclear for any other classifications.

### Statistical analysis

We summarised the total number of participants and number of events in each dataset, and the overall numbers available for validating each model.

#### Missing data

We could validate the predictive performance of a model only when the values of all its predictors were available for participants in at least one IPD dataset, i.e. in datasets where none of the predictors was systematically missing (unavailable for all participants). In such datasets, when data were missing for predictors and outcomes in some participants (‘partially missing data’), we used a 3-stage approach. First, where possible, we filled in the actual value that was missing using knowledge of the study’s eligibility criteria or by using other available data in the same dataset. For example, replacing nulliparous = 1 for all individuals in a dataset if only nulliparous women were eligible for inclusion. Secondly, after preliminary comparison of other datasets with the information, we used second trimester information in place of missing first trimester information. For example, early second trimester values of body mass index (BMI) or mean arterial pressure (MAP) were used if the first trimester values were missing. Where required, we reclassified into categories. Women of either Afro-Caribbean or African-American origin were classified as Black, and those of Indian or Pakistani origin as Asian. Thirdly, for any remaining missing values, we imputed all partially missing predictor and outcome values using multiple imputation by chained equations (MICE) [17, 18]. After preliminary checks comparing baseline characteristics in individuals with and without missing values for each variable, data were assumed to be missing at random (i.e. missingness conditional on other observed variables).

We conducted the imputations in each IPD dataset separately. This approach acknowledges the clustering of individuals within a dataset and retains potential heterogeneity across datasets. We generated 100 imputed datasets for each IPD dataset with any missing predictor or outcome values. In the multiple imputation models, continuous variables with missing values were imputed using linear regression (or predictive mean matching if skewed), binary variables were imputed using logistic regression, and categorical variables were imputed using multinomial logistic regression. Complete predictors were also included in the imputation models as auxiliary variables. To retain congeniality between the imputation models and predictive models [19], the scale used to impute the continuous predictors was chosen to match the prediction models. For example, pregnancy-associated plasma protein A (PAPP-A) was modelled on the log scale in many models and was therefore imputed as log(PAPP-A). We undertook imputation checks by looking at histograms, summary statistics, and tables of values across imputations, as well as by checking the trace plots for convergence issues.

#### Evaluating predictive performance of models

For each model that we could validate, we applied the model equation to each individual *i* in each (imputed) dataset. For each prediction model, we summarised the overall distribution of the linear predictor values for each dataset using the median, interquartile range, and full range, averaging statistics across imputations where necessary [20].

We examined the predictive performance of each model separately, using measures of discrimination and calibration, firstly in the IPD for each available dataset and then at the meta-analysis level. We assessed model discrimination using the *C*-statistic with a value of 1 indicating perfect discrimination and 0.5 indicating no discrimination beyond chance [21]. Good values of the *C*-statistic are hard to define, but we generally considered *C*-statistic values of 0.6 to 0.75 as moderate discrimination [22]. Calibration was assessed using the calibration slope (ideal value = 1, slope < 1 indicates overfitting, where predictions are too extreme) and the calibration-in-the-large (ideal value = 0). For each dataset containing over 100 outcome events, we also produced calibration plots to visually compare observed and predicted probabilities when there were enough events to categorise participants into 10 risk groups. These plots also included a lowess smoothed calibration curve over all individuals.

Where data had been imputed in a particular IPD dataset, the predictive performance measures were calculated in each of the imputed datasets, and then Rubin’s rules were applied to combine statistics (and corresponding standard errors) across imputations [20, 23, 24].

When it was possible to validate a model in multiple cohorts, we summarised the performance measures across cohorts using a random-effects meta-analysis estimated using restricted maximum likelihood (for each performance measure separately) [25, 26]. Summary (average) performance statistics were reported with 95% confidence intervals (derived using the Hartung-Knapp-Sidik-Jonkman approach as recommended) [27, 28]. We also reported the estimate of between-study heterogeneity (*τ*^2^) and the proportion of variability due to between-study heterogeneity (*I*^2^). Where there were five or more cohorts in the meta-analysis, we also reported the approximate 95% prediction interval (using the *t*-distribution to account for uncertainty in *τ*) [29]. We only reported the model performance in individual cohorts if the total number of events was over 100. We also compared the performance of the models in the same validation cohort where possible. We used forest plots to show a model’s performance in multiple datasets and to compare the average performance (across datasets) of multiple models.

A particular challenge is to predict pre-eclampsia in nulliparous women as they have no history from prior pregnancies (which are strong predictors); therefore, we also conducted a subgroup analysis in which we assessed the performance of the models in only nulliparous women from each study.

#### Decision curve analysis

For each pre-eclampsia outcome (any, early, or late onset), we compared prediction models using decision curve analysis [30, 31]. Decision curves show the net benefit (i.e. benefit versus harm) over a range of clinically relevant threshold probabilities. The model with the greatest net benefit for a particular threshold is considered to have the most clinical value. For this investigation, we chose the IPD that was most frequently used in the external validation of the prediction models and which allowed multiple models to be compared in the same IPD (thus enabling a direct, within-dataset comparison of the models).

All statistical analyses were performed using Stata MP Version 15. TRIPOD guidelines were followed for transparent reporting of risk prediction model validation studies [32, 33]. Additional details on the missing data checks, performance measures, meta-analysis, and decision curves are given in Supplementary Methods, Additional file 1 [20, 26, 34–45].

## Results

**Fig. 1:**
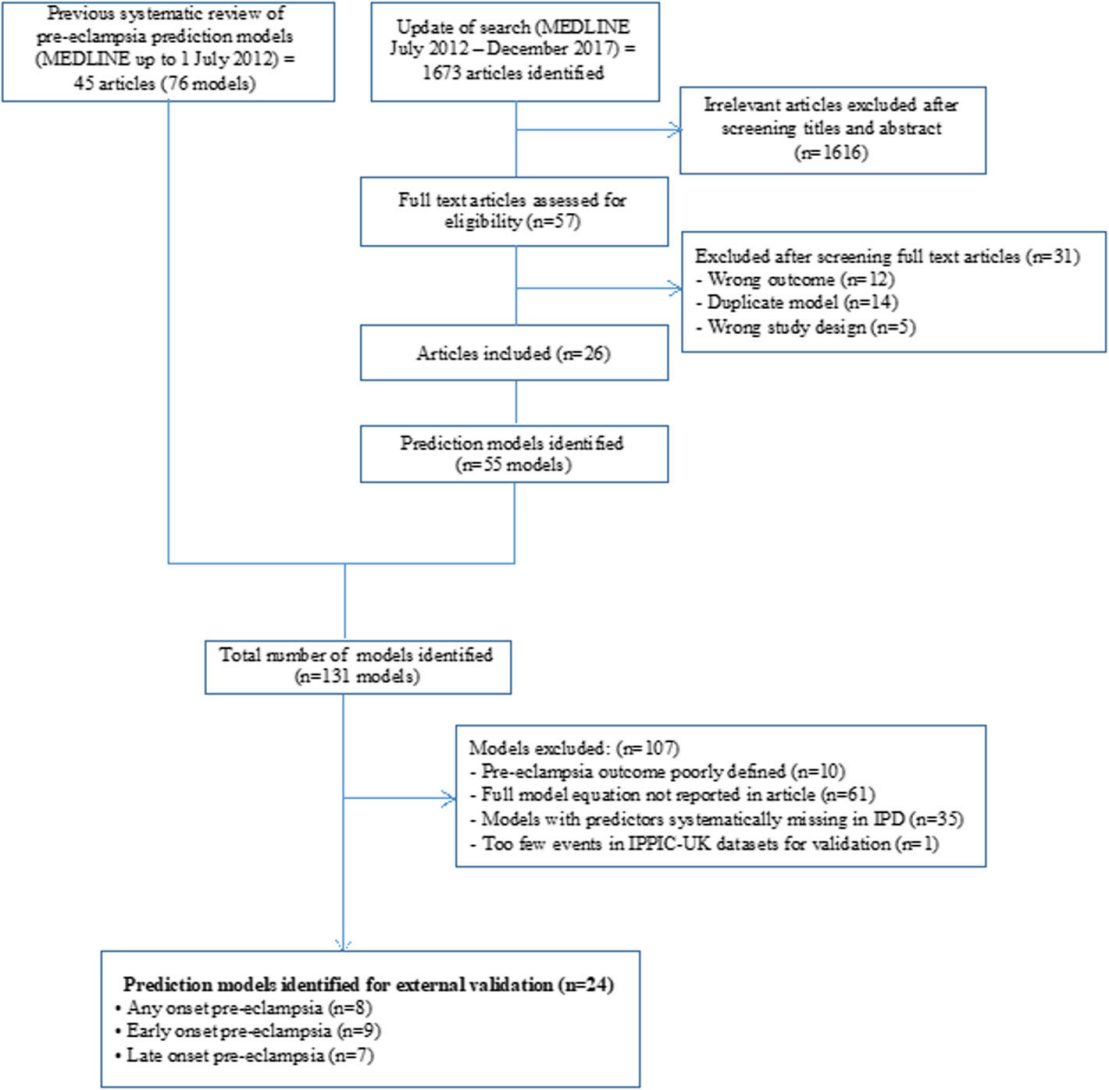
Identification of prediction models for validation in IPPIC-UK cohorts

Of the 131 models published on prediction of pre-eclampsia, only 67 reported the full model equation needed for validation (67/131, 51%) (Supplementary Table S3, Additional file 1). Twenty-four of these 67 models (24/67, 36%) met the inclusion criteria for external validation in the IPD datasets (Table 1) [35, 46–56], and the remaining models (43/67, 64%) did not meet the criteria due to the required predictor information not being available in the IPD datasets (Fig. 1).

**Table 1:** Pre-eclampsia prediction model equations externally validated in the IPPIC-UK cohorts

Model no.	Author (year)	Predictor category	Prediction model equation for linear predictor (LP)
**Trimester 1 any-onset pre-eclampsia models**			
1	Plasencia 2007a	Clinical characteristics	LP = − 6.253 + 1.432(if Afro-Caribbean ethnicity) + 1.465(if mixed ethnicity) + 0.084(BMI) + 0.81(if woman’s mother had PE) − 1.539(if parous without previous PE) + 1.049(if parous with previous PE)
2	Poon 2008	Clinical characteristics	LP = − 6.311 + 1.299(if Afro-Caribbean ethnicity) + 0.092(BMI) + 0.855(if woman’s mother had PE) − 1.481(if parous without previous PE) + 0.933(if parous with previous PE)
3	Wright 2015a*	Clinical characteristics	Mean gestational age at delivery with PE = 54.3637 − 0.0206886(age, years - 35, if age ≥ 35) + 0.11711(height, cm - 164) − 2.6786(if Afro-Caribbean ethnicity) − 1.129(if South Asian ethnicity) − 7.2897(if chronic hypertension) − 3.0519(if systemic lupus erythematosus or antiphospholipid syndrome) − 1.6327(if conception by in vitro fertilisation) − 8.1667(if parous with previous PE) + 0.0271988(if parous with previous PE, previous gestation in weeks - 24)^2^ − 4.335(if parous with no previous PE) − 4.15137651(if parous with no previous PE, interval between pregnancies in years)^−1^ + 9.21473572(if parous with no previous PE, interval between pregnancies in years)^−0.5^ − 0.0694096(if no chronic hypertension, weight in kg – 69) − 1.7154(if no chronic hypertension and family history of PE) − 3.3899(if no chronic hypertension and diabetes mellitus type 1 or 2)
4	Baschat 2014a	Clinical characteristics and biochemical markers	LP = − 8.72 + 0.157 (if nulliparous) + 0.341(if history of hypertension) + 0.635(if prior PE) + 0.064(MAP) − 0.186(PAPP-A, Ln MoM)
5	Goetzinger 2010	Clinical characteristics and biochemical markers	LP = − 3.25 + (0.51(if PAPP-A < 10th percentile) + 0.93(if BMI > 25) + 0.94(if chronic hypertension) + 0.97(if diabetes) + 0.61(if African American ethnicity)
6	Odibo 2011a	Clinical characteristics and biochemical markers	LP = − 3.389 − 0.716(PAPP-A, MoM) + 0.05(BMI) + 0.319(if black ethnicity) + 1.57(if history of chronic hypertension)
7	Odibo 2011b	Clinical characteristics and ultrasound markers	LP = − 3.895 − 0.593(mean uterine PI) + 0.944(if pre-gestational diabetes) + 0.059(BMI) + 1.532(if history of chronic hypertension)
**Trimester 2 any-onset pre-eclampsia models**			
8	Yu 2005a	Clinical characteristics and ultrasound markers	LP = 1.8552 + 5.9228(mean uterine PI)^−2^ − 14.4474(mean uterine PI)^−1^ − 0.5478(if smoker) + 0.6719(bilateral notch) + 0.0372(age) + 0.4949(if black ethnicity) + 1.5033(if history of PE) − 1.2217(if previous term live birth) + 0.0367(T2 BMI)
**Trimester 1 early-onset pre-eclampsia models**			
9	Baschat 2014b	Clinical characteristics	LP = − 5.803 + 0.302(if history of diabetes) + 0.767 (if history of hypertension) + 0.00948(MAP)
10	Crovetto 2015a	Clinical characteristics	LP = − 5.177 + (2.383 if black ethnicity) − 1.105(if nulliparous) + 3.543(if parous with previous PE) + 2.229(if chronic hypertension) + 2.201(if renal disease)
11	Kuc 2013a	Clinical characteristics	LP = − 6.790 − 0.119(maternal height, cm) + 4.8565(maternal weight, Ln kg) + 1.845(if nulliparous) + 0.086(maternal age, years) + 1.353(if smoker)
12	Plasencia 2007b	Clinical characteristics	LP = − 6.431 + 1.680(if Afro-Caribbean ethnicity) + 1.889(if mixed ethnicity) + 2.822(if parous with previous PE)
13	Poon 2010a	Clinical characteristics	LP = − 5.674 + 1.267(if black ethnicity) + 2.193(if history of chronic hypertension) − 1.184(if parous without previous PE) + 1.362(if parous with previous PE) + 1.537(if conceived with ovulation induction)
14	Scazzocchio 2013a	Clinical characteristics	LP = − 7.703 + 0.086(BMI) + 1.708(if chronic hypertension) + 4.033(if renal disease) + 1.931(if parous with previous PE) + 0.005(if parous with no previous PE)
15	Wright 2015b*	Clinical characteristics	Same as model 3
16	Poon 2009a	Clinical characteristics and biochemical markers	LP = − 6.413 − 3.612 (PAPP-A, Ln MoM) + 1.803(if history of chronic hypertension) + 1.564(if black ethnicity) − 1.005(if parous without previous PE) + 1.491(if parous with previous PE)
**Trimester 2 early-onset pre-eclampsia models**			
17	Yu 2005b	Clinical characteristics and ultrasound markers	LP = − 9.81223 + 2.10910(mean uterine PI)^3^ − 1.79921(mean uterine PI)^3^ + 1.059463(if bilateral notch)
**Trimester 1 late-onset pre-eclampsia models**			
18	Crovetto 2015b	Clinical characteristics	LP = − 5.873 − 0.462(if white ethnicity) + 0.109(BMI) − 0.825(if nulliparous) + 2.726(if parous with previous PE) + 1.956(if chronic hypertension) − 0.575(if smoker)
19	Kuc 2013b	Clinical characteristics	LP = − 14.374 + 2.300(maternal weight, Ln kg) + 1.303(if nulliparous) + 0.068(maternal age, years)
20	Plasencia 2007c	Clinical characteristics	LP = − 6.585 + 1.368(if Afro-Caribbean ethnicity) + 1.311(if mixed ethnicity) + 0.091(BMI) + 0.960(if woman’s mother had PE) − 1.663(if parous without previous PE)
21	Poon 2010b	Clinical characteristics	LP = − 7.860 + 0.034(maternal age, years) + 0.096(BMI) + 1.089(if black ethnicity) + 0.980(if Indian or Pakistani ethnicity) + 1.196(if mixed ethnicity) + 1.070(if woman’s mother had PE) − 1.413(if parous without previous PE) + 0.780(if parous with previous PE)
22	Scazzocchio 2013b	Clinical characteristics	LP = 6.135 + 2.124(if previous PE) + 1.571(if chronic hypertension) + 0.958(if diabetes) + 1.416(if thrombophilic condition) − 0.487(if multiparous) + 0.093(BMI)
23	Poon 2009b	Clinical characteristics and biochemical markers	LP = − 6.652 − 0.884(PAPP-A, Ln MoM) + 1.127(if family history of PE) + 1.222(if black ethnicity) + 0.936(if Indian or Pakistani ethnicity) + 1.335(if mixed ethnicity) + 0.084(BMI) − 1.255(if parous without previous PE) + 0.818(if parous with previous PE)
**Trimester 2 late-onset pre-eclampsia models**			
24	Yu 2005c	Clinical characteristics and ultrasound markers	LP = 0.7901 + 5.1473(mean uterine PI)^−2^ − 12.5152(mean uterine PI)^−1^ − 0.5575(if smoker) + 0.5333(if bilateral notch) + 0.0328(age) + 0.4958(if black ethnicity) + 1.5109(if history of PE) + 1.1556(if previous term live birth) + 0.0378(BMI)

* The model for ‘mean gestational age at delivery with PE’ assumes a normal distribution with the predicted mean gestational age and SD=6.8833. The risk of delivery with PE is then calculated as the area under the normal curve between 24 weeks and either 42 weeks for any onset PE (model 3) or 34 weeks for early-onset PE (model 14). For more detail see Wright et al., 2015.

### Characteristics and quality of the validation cohorts

IPD from 11 cohorts contained within the IPPIC network contained relevant predictors and outcomes that could be used to validate at least one of the 24 prediction models. Four of the 11 validation cohorts were prospective observational studies (Allen 2017, POP, SCOPE, and Velauthar 2012) [36, 37, 45], four were nested within randomised trials (Chappell 1999, EMPOWAR, Poston 2006, and UPBEAT) [39–42], and three were from prospective registry datasets (ALSPAC, AMND, and St George’s) [38, 43, 44, 57]. Six cohorts included pregnant women with high and low risk of pre-eclampsia [37, 38, 43–45], four included high-risk women only [39–42], and one included low-risk women only [36]. Two of the 11 cohorts (SCOPE, POP) included only nulliparous women with singleton pregnancies who were at low risk [36] and at any risk of pre-eclampsia [45]. In the other 9 cohorts, the proportion of nulliparous women ranged from 43 to 65%. Ten of the 11 cohorts reported on any-, early-, and late-onset pre-eclampsia, while one had no women with early-onset pre-eclampsia [40]. The characteristics of the validation cohorts and a summary of the missing data for each predictor and outcome are provided in Supplementary Tables S4, S5, and S6 (Additional file 1), respectively.

A fifth of all validation cohorts (2/11, 18%) were classed as having an overall low risk of bias for all three PROBAST domains of participant selection, predictor evaluation, and outcome assessment. Seven (7/11, 64%) had low risk of bias for participant selection domain, and ten (10/11, 91%) had low risk of bias for predictor assessment, while one had an unclear risk of bias for that domain. For outcome assessment, half of all cohorts had low risk of bias (5/11, 45%) and it was unclear in the rest (6/11, 55%) (Supplementary Table S7, Additional file 1).

### Characteristics of the validated models

All of the models we validated were developed in unselected populations of high- and low-risk women. About two thirds of the models (63%, 15/24) included only clinical characteristics as predictors [35, 46, 47, 49, 51–53, 55], five (21%) included clinical characteristics and biomarkers [46, 48, 50, 54], and four (17%) included clinical characteristics and ultrasound markers [50, 56]. Most models predicted the risk of pre-eclampsia using first trimester predictors (21/24, 88%), and three using first and second trimester predictors (13%). Eight models predicted any-onset pre-eclampsia, nine early-onset, and seven predicted late-onset pre-eclampsia (Table 1). The sample size of only a quarter of the models (25%, 6/24) [35, 47, 48, 56] was considered adequate, based on having at least 10 events per predictor evaluated to reduce the potential for model overfitting.

### External validation and meta-analysis of predictive performance

We validated the predictive performance of each of the 24 included models in at least one and up to eight validation cohorts. The distributions of the linear predictor and the predicted probability are shown for each model and validation cohort in Supplementary Table S8 (Additional file 1). Performance of models is given for each cohort separately (including smaller datasets) in Supplementary Table S9 (Additional file 1).

#### Performance of models predicting any-onset pre-eclampsia

Two clinical characteristics models (Plasencia 2007a; Poon 2008) with predictors such as ethnicity, family history of pre-eclampsia, and previous history of pre-eclampsia showed reasonable discrimination in validation cohorts with summary *C*-statistics of 0.69 (95% CI 0.53 to 0.81) for both models (Table 2). The models were potentially overfitted (summary calibration slope < 1) indicating extreme predictions compared to observed events, with wide confidence intervals, and large heterogeneity in discrimination and calibration (Table 2). The third model (Wright 2015a) included additional predictors such as history of systemic lupus erythematosus, anti-phospholipid syndrome, history of in vitro fertilisation, chronic hypertension, and interval between pregnancies, and showed less discrimination (summary *C*-statistic 0.62, 95% CI 0.48 to 0.75), with observed overfitting (summary calibration slope 0.64) (Table 2).

**Table 2:** Summary estimates of predictive performance for each model across validation cohorts

Model no.	Type of predictors	Author (year)	No. of validation cohorts	Total no. of women	Total events	Summary estimate of performance statistic (95% CI), measures of heterogeneity (***I***^**2**^, ***τ***^**2**^)		
						***C***-statistic^**+**^	Calibration slope	Calibration-in-the-large
**Any-onset pre-eclampsia**								
***Trimester 1 models***								
1	Clinical	Plasencia 2007a	3	3257	102	0.69 (0.53, 0.81) *I*^2^ = 1%, *τ*^2^ = 0.001	0.69 (− 0.03, 1.41) *I*^2^ = 45%, *τ*^2^ = 0.035	0.14 (− 1.47, 1.76) *I*^2^ = 91%, *τ*^2^ = 0.380
2		Poon 2008	3	3257	102	0.69 (0.53, 0.81) *I*^2^ = 3%, *τ*^2^ = 0.002	0.72 (− 0.03, 1.46) *I*^2^ = 45%, *τ*^2^ = 0.037	0.002 (− 1.65, 1.66) *I*^2^ = 92%, *τ*^2^ = 0.402
3		Wright 2015a	3	1916	76	0.62 (0.48, 0.75) *I*^2^ = 0%, *τ*^2^ = 0	0.64 (− 0.18, 1.47) *I*^2^ = 0%, *τ*^2^ = 0	0.95 (− 1.13, 3.03) *I*^2^ = 93%, *τ*^2^ = 0.640
4	Clinical and biochemical markers	Baschat 2014a	2	5257	287	0.71 (0.47, 0.87) *I*^2^ = 0%, *τ*^2^ = 0	1.24 (0.00, 2.48) *I*^2^ = 0%, *τ*^2^ = 0	− 0.43 (− 14.4, 13.55) *I*^2^ = 98%, *τ*^2^ = 2.382
5		Goetzinger 2010	3	6811	343	0.66 (0.30, 0.90) *I*^2^ = 93%, *τ*^2^ = 0.315	1.124 (− 0.60, 2.84) *I*^2^ = 76%, *τ*^2^ = 0.356	− 0.97 (− 3.04, 1.11) *I*^2^ = 97%, *τ*^2^ = 0.667
6		Odibo 2011a	3	59,892	1774	0.72 (0.51, 0.86) *I*^2^ = 90%, *τ*^2^ = 0.101	1.16 (0.24, 2.08) *I*^2^ = 93%, *τ*^2^ = 0.104	− 0.79 (− 2.62, 1.04) *I*^2^ = 99%, *τ*^2^ = 0.511
7	Clinical and ultrasound markers	Odibo 2011b	1	1145	28	0.53 (0.39, 0.66)	0.28 (− 0.64, 1.20)	− 0.52 (− 0.91, − 0.13)
***Trimester 2 models***								
8	Clinical and ultrasound markers	Yu 2005a	1	4212	273	0.61 (0.57 to 0.65)	0.08 (0.01 to 0.14)	Not estimable
**Early-onset pre-eclampsia**								
***Trimester 1 models***								
9	Clinical	Baschat 2014b	5	22,781	204	0.68 (0.62, 0.73) *I*^2^ = 0%, *τ*^2^ = 0	2.04 (0.56, 3.52) *I*^2^ = 69%, *τ*^2^ = 0.692	− 0.10 (− 1.70 to 1.49) *I*^2^ = 97%, *τ*^2^ = 1.535
10		Crovetto 2015a	3^#^	6424	21	0.58 (0.21, 0.88) *I*^2^ = 69%, *τ*^2^ = 0.288	0.64 (− 4.01, 5.29) *I*^2^ = 81%, *τ*^2^ = 0.217	− 0.58 (− 4.97, 3.81) *I*^2^ = 95%, *τ*^2^ = 2.925
11		Kuc 2013a	6	212,038	1449	0.66 (0.61, 0.71) *I*^2^ = 32%, *τ*^2^ = 0.011	0.42 (0.29, 0.55) *I*^2^ = 33%, *τ*^2^ = 0.004	− 4.33 (− 5.41, − 3.25) *I*^2^ = 99%, *τ*^2^ = 0.946
12		Plasencia 2007b	4^#^	6740	27	0.49 (0.43, 0.55) *I*^2^ = 38%, *τ*^2^ = 0.005	0.51 (− 2.05, 3.08) *I*^2^ = 0%, *τ*^2^ = 0	0.47 (− 0.80, 1.74) *I*^2^ = 74%, *τ*^2^ = 0.452
13		Poon 2010a	3	6424	21	0.64 (0.31, 0.87) *I*^2^ = 34%, *τ*^2^ = 0.105	0.99 (0.02, 1.96) *I*^2^ = 0%, *τ*^2^ = 0	− 1.09 (− 4.89, 2.70) *I*^2^ = 93%, *τ*^2^ = 2.175
14		Scazzocchio 2013a	3	6424	21	0.74 (0.37, 0.93) *I*^2^ = 14%, *τ*^2^ = 0.057	0.75 (0.14, 1.36) *I*^2^ = 0%, *τ*^2^ = 0	− 0.70 (− 3.89, 2.49) *I*^2^ = 90%, *τ*^2^ = 1.481
15		Wright 2015b	2	1332	9	0.74 (0.04, 1.00) *I*^2^ = 0%, *τ*^2^ = 0	0.92 (− 4.38, 6.22) *I*^2^ = 0%, *τ*^2^ = 0	0.28 (− 14.34, 14.90) *I*^2^ = 90%, *τ*^2^ = 2.395
16	Clinical and biochemical markers	Poon 2009a	1	4212	10	0.74 (0.51, 0.89)	0.45 (0.21, 0.69)	− 2.67 (− 3.35, − 1.99)
***Trimester 2 models***								
17	Clinical and ultrasound markers	Yu 2005b	1	4212	10	0.91 (0.83, 0.95)	0.56 (0.29, 0.82)	2.47 (1.72, 3.23)
**Late-onset pre-eclampsia**								
***Trimester 1 models***								
18	Clinical	Crovetto 2015b	5	7785	384	0.63 (0.46, 0.78) *I*^2^ = 87%, *τ*^2^ = 0.264	0.56 (− 0.01 to 1.13) *I*^2^ = 92%, *τ*^2^ = 0.179	− 0.05 (− 1.65, 1.55) *I*^2^ = 98%, *τ*^2^ = 1.615
19		Kuc 2013b	8	213,532	5716	0.62 (0.57, 0.67) *I*^2^ = 87%, *τ*^2^ = 0.025	0.66 (0.50, 0.82) *I*^2^ = 60%, *τ*^2^ = 0.007	− 1.91 (− 2.24, − 1.59) *I*^2^ = 98%, *τ*^2^ = 0.124
20		Plasencia 2007c	3	3257	90	0.67 (0.54, 0.78) *I*^2^ = 0%, *τ*^2^ = 0	0.61 (0.04, 1.18) *I*^2^ = 14%, *τ*^2^ = 0.008	0.20 (− 1.11, 1.52) *I*^2^ = 85%, *τ*^2^ = 0.234
21		Poon 2010b	3	3257	90	0.65 (0.48, 0.79) *I*^2^ = 25%, *τ*^2^ = 0.020	0.57 (0.08, 1.05) *I*^2^ = 0%, *τ*^2^ = 0	0.12 (− 1.59, 1.84) *I*^2^ = 91%, *τ*^2^ = 0.430
22		Scazzocchio 2013b	1	658	26	0.60 (0.48, 0.71)	0.56 (− 0.17, 1.29)	0.52 (0.13, 0.92)
23	Clinical and biochemical markers	Poon 2009b	1	1045	13	0.68 (0.55, 0.79)	0.80 (0.26, 1.34)	− 0.35 (− 0.90, 0.21)
***Trimester 2 models***								
24	Clinical and ultrasound markers	Yu 2005c	1	4212	263	0.61 (0.57, 0.64)	0.08 (0.05, 0.15)	Not estimable

# Number of validation cohorts is 2 for the calibration slope as it could not be estimated reliably in SCOPE (for models 10 and 12) or POP (for model 12), and was therefore excluded from the meta-analysis. + The C-statistic was pooled on the logit scale, therefore *I*^*2*^ is for logit(C-statistic).

**Fig. 2:**
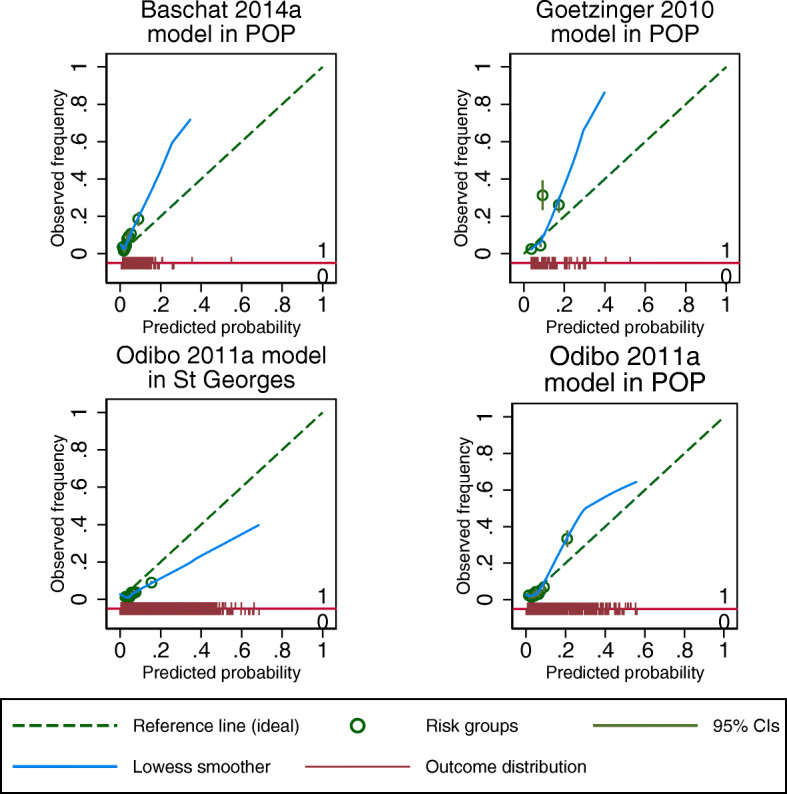
Calibration plots for clinical characteristic and biomarker models predicting any-onset pre-eclampsia (cohorts with ≥ 100 events)

The three models with clinical and biochemical predictors (Baschat 2014a; Goetzinger 2010; Odibo 2011a) showed moderate discrimination (summary *C*-statistics 0.66 to 0.72) (Table 2). We observed underfitting (summary calibration slope > 1) with predictions that do not span a wide enough range of probabilities compared to what was observed in the validation cohorts (Fig. 2). Amongst these three models, the Odibo 2011a model with ethnicity, BMI, history of hypertension, and PAPP-A as predictors showed the highest discrimination (summary *C*-statistic 0.72, 95% CI 0.51 to 0.86), with a summary calibration slope of 1.20 (95% CI 0.24 to 2.00) due to heterogeneity in calibration performance across the three cohorts.

When validated in individual cohorts, the Odibo 2011a model demonstrated better discrimination in the POP cohort of any risk nulliparous women (*C*-statistics 0.78, 95% CI 0.74 to 0.81) than in the St George’s cohort of all pregnant women (*C*-statistics 0.67, 95% CI 0.65 to 0.69). The calibration estimates for Odibo 2011a model in these two cohorts showed underfitting in the POP cohort (calibration slope 1.49, 95% CI 1.33 to 1.65) and reasonably adequate calibration in the St George’s cohort (slope 0.96, 95% CI 0.89 to 1.04). The calibration-in-the-large of the Odibo 2011a showed systematic overprediction in the St George’s cohort (− 0.90, 95% CI − 0.95 to − 0.85) and less so in the POP cohort with value close to 0. Both Baschat 2014a and Goetzinger 2010 models also showed moderate discrimination in the POP cohort with *C*-statistics ranging from 0.70 to 0.76. When validated in the POP cohort, the Baschat 2014a model systematically underpredicted risk with calibration-in-the-large (0.66, 95% CI 0.53 to 0.78) and less so for the Goetzinger 2010 model. One model (Yu 2005a) that included second trimester ultrasound markers and clinical characteristics had low discrimination (*C*-statistic 0.61, 95% CI 0.57 to 0.65) and poor calibration (slope 0.08, 95% CI 0.01 to 0.14), and was only validated in the POP cohort (Table 3).

**Table 3:** Predictive performance statistics for models in the individual IPPIC-UK cohorts with over 100 events

Model no.	Author (year)	Predictor	Sovio 2015 (4212 women)			Stirrup 2015 (54,635 women)			Ayorinde 2016 (136,635 women)			Poston 2006 (2422 women)			Fraser 2013 (14,344 women)		
			***C***-statistic (95% CI)	Calibration slope (95% CI)	CITL (95% CI)	***C***-statistic (95% CI)	Calibration slope (95% CI)	CITL (95% CI)	***C***-statistic (95% CI)	Calibration slope (95% CI)	CITL (95% CI)	***C***-statistic (95% CI)	Calibration slope (95% CI)	CITL (95% CI)	***C***-statistic (95% CI)	Calibration slope (95% CI)	CITL (95% CI)
**Any-onset pre-eclampsia models**																	
4	Baschat 2014a	Clinical and biochemical	0.71 (0.67, 0.74)	1.24 (1.03, 1.44)	0.66 (0.53, 0.78)												
5	Goetzinger 2010		0.76 (0.73, 0.80)	1.71 (1.50, 1.91)	− 0.07 (− 0.20, 0.05)												
6	Odibo 2011a		0.78 (0.74, 0.81)	1.49 (1.33, 1.65)	− 0.03 (− 0.16, 0.09)	0.67 (0.65, 0.69)	0.96 (0.89, 1.04)	− 0.90 (− 0.95, − 0.85)									
8	Yu 2005a	Clinical and ultrasound	0.61 (0.57, 0.65)	0.08 (0.01, 0.14)	Not estimable												
**Early-onset pre-eclampsia models**																	
9	Baschat 2014b	Clinical										0.67 (0.63, 0.72)	1.28 (0.90, 1.66)	1.80 (1.63, 1.97)			
11	Kuc 2013a					0.64 (0.59, 0.68)	0.34 (0.23, 0.46)	− 4.51 (− 4.67, − 4.35)	0.68 (0.67, 0.70)	0.47 (0.43, 0.51)	− 3.39 (− 3.45, − 3.33)						
**Late-onset pre-eclampsia models**																	
18	Crovetto 2015b	Clinical	0.78 (0.75, 0.81)	1.25 (1.12, 1.38)	1.31 (1.18, 1.44)												
19	Kuc 2013b		0.60 (0.56, 0.64)	0.67 (0.45, 0.89)	− 1.49 (− 1.61, − 1.36)	0.64 (0.62, 0.65)	0.63 (0.56, 0.70)	− 1.97 (− 2.03, − 1.92)	0.84 (0.64 to 0.94)	0.75 (0.45, 1.04)	− 1.44 (− 2.09, − 0.79)				0.66 (0.62, 0.70)	0.76 (0.55, 0.97)	− 1.57 (− 1.70, − 1.45)
24	Yu 2005c	Clinical and ultrasound	0.61 (0.57, 0.64)	0.08 (0.01, 0.15)	Not estimable												

CITL = Calibration-in-the-large

#### Performance of models predicting early-onset pre-eclampsia

We then considered the prediction of early-onset pre-eclampsia. The two clinical characteristics models, Baschat 2014b with predictors such as history of diabetes, hypertension, and mean arterial pressure [46], and Kuc 2013a model with maternal height, weight, parity, age, and smoking status [49], showed reasonable discrimination (summary *C*-statistics 0.68, 0.66, respectively) with minimal heterogeneity when validated in up to six datasets. The summary calibration was suboptimal with either under- or overfitting. When validated in individual cohorts (Poston 2006, St George’s, and AMND cohorts), the Kuc model showed moderate discrimination in the St George’s and AMND cohorts of unselected pregnant women with values ranging from 0.64 to 0.68, respectively. But the model was overfitted in both the cohorts (calibration slope 0.34 and 0.47) and systematically overpredicted the risks (calibration-in-the-large > 1). In the external cohort of obese pregnant women (Poston 2006), Baschat 2014b model showed moderate discrimination (*C*-statistic 0.67, 95% CI 0.63 to 0.72). There was some evidence that predictions did not span a wide enough range of probabilities and that the model systematically underpredicted the risks (Table 3).

The other six models were validated with a combined total of less than 50 events between the cohorts [35, 47, 51, 52, 55]. Of these, the clinical characteristics models of Scazzocchio 2013a and Wright 2015b, and the clinical and biochemical marker-based model of Poon 2009a showed promising discrimination (summary *C*-statistic 0.74), but with imprecise estimates indicative of the small sample size in the validation cohorts. All three models were observed to be overfitted (summary calibration slopes ranging from 0.45 to 0.91), though again confidence intervals were wide. The second trimester Yu 2005b model with ultrasound markers and clinical characteristics was validated in one cohort with 10 events, resulting in very imprecise estimates but still indicative of the model being overfitted (calibration slope 0.56, 95% CI 0.29 to 0.82).

#### Performance of models predicting late-onset pre-eclampsia

Of the five clinical characteristics models, four (Crovetto 2015b, Kuc 2010b, Plasencia 2007c, Poon 2010b) were validated across cohorts. The models showed reasonable discrimination with summary *C*-statistics ranging between 0.62 and 0.67 [47, 49, 51, 52]. We observed overfitting (summary calibration slope 0.56 to 0.66) with imprecision except for the Kuc 2013b model. The models appeared to either systematically underpredict (Plasencia 2007c, Poon 2010b) or overpredict (Crovetto 2015b, Kuc 2013b), with imprecise calibration-in-the-large estimates. There was moderate to large heterogeneity in both discrimination and calibration measures.

When validated in the POP cohort of nulliparous women, the Crovetto 2015b model with predictors such as maternal ethnicity, parity, chronic hypertension, smoking status, and previous history of pre-eclampsia showed good discrimination (*C*-statistic 0.78, 95% CI 0.75 to 0.81) but with evidence of some underfitting (calibration slope 1.25, 95% CI 1.10 to 1.38); the model also systematically underpredicted the risks (calibration-in-the-large 1.31, 95% CI 1.18 to 1.44). The corresponding performance of the Kuc 2010b model in the POP cohort showed low discrimination (*C*-statistic 0.60, 95% CI 0.56 to 0.64) and calibration (calibration slope 0.67, 95% CI 0.45 to 0.89). In the ALSPAC, St George’s, and AMND unselected pregnancy cohorts, the Kuc 2010b model showed varied discrimination with *C*-statistics ranging from 0.64 to 0.84, but with overfitting (calibration slope < 1) and systematic overprediction (calibration-in-the-large − 1.97, 95% CI − 1.57 to − 1.44). In the POP cohort, the Yu 2005c model with clinical and second trimester ultrasound markers had a *C*-statistic of 0.61 (95% CI 0.57 to 0.64) with severe overfitting (calibration slope 0.08, 95% CI 0.01 to 0.15).

Supplementary Table S10 (Additional file 1) shows the performance of the models in nulliparous women only in the IPPIC-UK datasets and in the POP cohort only separately.

#### Heterogeneity

Where it was possible to estimate it, heterogeneity across studies varied from small (e.g. Plasencia 2007a and Poon 2008 models had *I*^2^ ≤ 3%, *τ*^2^ ≤ 0.002) to large heterogeneity (e.g. Goetzinger 2010 and Odibo 2011a models had *I*^2^ ≥ 90%, *τ*^2^ ≥ 0.1) for the *C*-statistic (on the logit scale), and moderate to large heterogeneity in the calibration slope for about two thirds (8/13, 62%) of all models validated in datasets with around 100 events in total. All models validated in multiple IPD datasets had high levels of heterogeneity in calibration-in-the-large performance. For the majority of models validated in cohorts with a combined event size of around 100 events in total (9/13, 69%), the summary calibration slope was less than or equal to 0.7 suggesting a general concern of overfitting in the model development (as ideal value is 1, and values < 1 indicate predictions are too extreme). The exceptions to this were Baschat 2014a, Goetzinger 2010, and Odibo 2011a models (for any-onset pre-eclampsia) and Baschat 2014b (for early-onset pre-eclampsia).

### Decision curve analysis

**Fig. 3:**
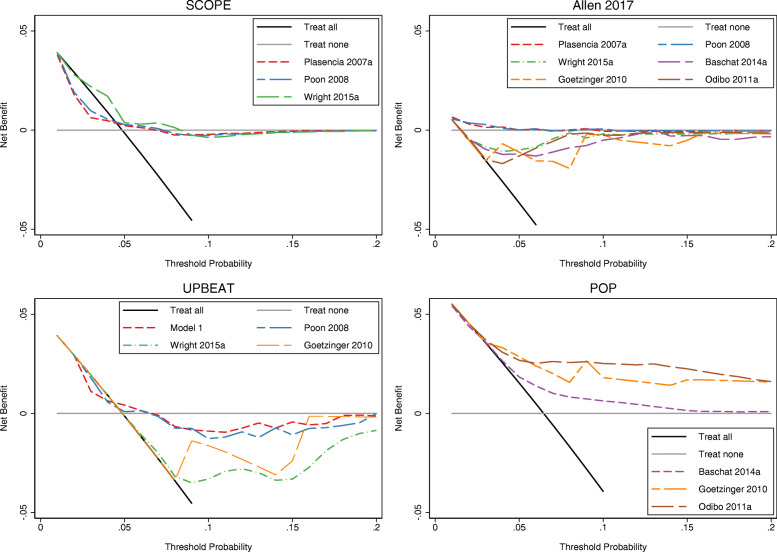
Decision curves for models of any-onset pre-eclampsia

We compared the clinical utility of models for any-onset pre-eclampsia in SCOPE (3 models), Allen 2017 (6 models), UPBEAT (4 models), and POP cohorts (3 models) as they allowed us to compare more than one model. Of the three models validated in the POP cohort [46, 48, 50], the Odibo 2011a model had the highest clinical utility for a range of thresholds for predicting any-onset pre-eclampsia (Fig. 3). But this net benefit was not observed either for Odibo 2011a or for other models when validated in the other cohorts. Decision curves for early- and late-onset pre-eclampsia models are given in Supplementary Figure S1 and S2 (Additional file 1), respectively. These showed that there was little opportunity for net benefit of the early-onset pre-eclampsia prediction models, primarily because of how rare the condition is. For late-onset pre-eclampsia, the models showed some net benefit across a very narrow range of threshold probabilities.

## Discussion

### Summary of findings

Of the 131 prediction models developed for predicting the risk of pre-eclampsia, only half published the model equation that is necessary for others to externally validate these models, and of those remaining, only 25 included predictors available to us in the datasets of the validation cohorts. One model could not be validated because of too few events in the validation cohorts. In general, models moderately discriminated between women who did and did not develop any-, early-, or late-onset pre-eclampsia. The performance did not appear to vary noticeably according to the type of predictors (clinical characteristics only; additional biochemical or ultrasound markers) or the trimester. Overall calibration of predicted risks was generally suboptimal. In particular, the summary calibration slope was often much less than 1, suggesting that the developed models were overfitted to their development dataset and thus do not transport well to new populations. Even for those with promising summary calibration performance (e.g. summary calibration slopes close to 1 from the meta-analysis), we found large heterogeneity across datasets, indicating that the calibration performance of the models is unlikely to be reliable across all UK settings represented by the validation cohorts. Some models showed promising performance in nulliparous women, but this was not observed in other populations.

### Strengths and limitations

To our knowledge, this is the first IPD meta-analysis to externally validate existing prediction models for pre-eclampsia. Our comprehensive search identified over 130 published models, illustrating the desire for risk prediction in this field, but also the confusion about which models are reliable. The global IPPIC Network brought together key researchers involved in this field, and their cohorts provided access to the largest IPD on prediction of pregnancy complications. We evaluated whether any of the identified models demonstrated good predictive performance in the UK health system, both on average and within individual cohorts. Access to raw data meant that we could exclude ineligible women, account for timing of predictor measurement and outcome, and increase the sample size for rare outcomes such as early-onset pre-eclampsia.

We could only validate 24 of the 131 published pre-eclampsia prediction models and were restricted by poor reporting of published models, as well as the unavailability of predictors used in some reported models within our IPD. It is possible that a better performing model exists which we have been unable to validate. However, the issue of missing predictors may also reflect the availability of these predictors in routine clinical practice, and the inconvenience in their measurement, highlighting the need for a practical prediction model with easy to measure and commonly reported variables [58].

We limited our validation to UK datasets to reduce the heterogeneity arising from outcome definitions and variations in management. Despite this, often considerable heterogeneity remained in predictive performance. Direct comparison of the prediction models is difficult due to different datasets contributing towards the validation of each model.

### Comparison to existing studies

Currently, none of the published models on pre-eclampsia has been recommended for clinical practice. We consider the following issues to contribute to this phenomenon. Firstly, most of the models have never been externally validated, and their performance in other populations is unknown [6, 37, 59–61]. Secondly, even when validated, the findings are limited by the relatively small numbers of events in the validation cohort to draw robust conclusions, for example about calibration performance. Recently, first trimester models for any pre-eclampsia comprising of easily available predictors were validated in two separate Dutch cohorts in line with current recommendations. Both validation cohorts comprised of less than 100 events each, which is recommended as the minimum sample size required [6]. Discrimination of these models was moderate and similar to what we observed. Most models showed overfitting and systematic overprediction of the risks. The clinical utility of the best performing models showed net benefit over a narrow range of probabilities. Thirdly, there is fatigue amongst the research community and the clinicians due to the vast numbers of prediction models that have been published with various combinations and permutations of predictor variables, often in overlapping populations without external validation [35, 51, 53, 54, 62–79].

Fourthly, many models have been developed by considering them as a ‘screening test’ for pre-eclampsia, similar to the approach used in Down syndrome screening with biomarkers. In addition to the lack of information on multiple of the median (MoM) values in validating cohorts, such an approach has inherent limitations. The models’ performances are reported in terms of detection rate (sensitivity) for a specific false positive rate of 10% [35, 51, 54, 63–66, 68–71, 75, 77–79], but unlike diagnostic tests (where focus is on sensitivity and specificity), when predicting future outcomes it is more important to provide absolute risk predictions, potentially across the whole spectrum of risk (from 0 to 1) [80]. Such risk predictions then guide patient counselling, shared clinical decision-making, and personalisation of healthcare. As such, calibration of such risk predictions must be checked. In population-based cohorts, only a small proportion of individuals are at high risk of pre-eclampsia, with a preponderance of those at low or very low risk. However, the performance of many models continues to be evaluated and compared solely on the basis of their discrimination ability, with calibration ignored [81].

In the recent ASPRE (Combined Multimarker Screening and Randomized Patient Treatment with Aspirin for Evidence-Based Preeclampsia Prevention) trial [82], aspirin significantly reduced the risk of pre-eclampsia in women stratified for high risk of preterm pre-eclampsia using the prediction model by Akolekar 2013 [62]. In the control group, 4.3% of women were considered to have preterm pre-eclampsia against the 7.6% expected to be identified by the model. The discrimination of the model was published recently, and its calibration reported in two separate datasets [83]. The so-called competing risks model appears to have exceptional performance and very high discrimination (> 0.8) when validated in datasets from a standardised population akin to that used for model development. While this is laudable, caution is needed. The model showed evidence of some problems with calibration-in-the-large and did not examine heterogeneity in calibration performance across centres. Even if all centres across the UK use the same standardisation as the SPREE studies (in terms of timing and methods of predictor measurement), there may still be heterogeneity in the model performance, for example if the baseline risk of pre-eclampsia varied across centres. Therefore, before widespread uptake or implementation of this model, detailed exploration of the performance in a wide range of realistic settings of application is needed, including decision curve analyses. We were not able to validate this model in IPPIC-UK datasets due to lack of information on predictors, and other information needed to calculate the MoMs.

### Relevance to clinical practice

A clinically useful prediction model should be able to accurately identify women who are at risk of pre-eclampsia in all healthcare settings that the model will be used. There is no evidence from this IPD meta-analysis that, for the subset of published models we could evaluate, any model is applicable for use across all populations within UK healthcare setting. In particular, the poor observed calibration and the large heterogeneity across different datasets suggest that the subset of models are not robust enough for widespread use. It is likely that the predictive performance of the models would be improved by recalibration to particular settings and populations, for which local data are needed. This may not be practical in practice.

### Recommendations for further research

A major issue is that, based on the subset of models evaluated, existing prediction models in the pre-eclampsia field appear to suffer from calibration slopes < 1 in new data, which is likely to reflect overfitting when developing the model. This is known to be a general problem for the prediction model field in other disease areas [84]. To reduce the impact of overfitting, predictor effects might be corrected by shrinking the predictor effects (i.e. using penalisation techniques during model development—a similar concept is regression to the mean) [85–88] and performing appropriate internal validation (e.g. using bootstrapping) [89]. Furthermore, to improve the overall calibration across settings, the baseline risk (through the intercept) may need to be tailored to the different settings. This can, for instance, be achieved by comparing the ‘local’ outcome incidence with the reported incidence from the original development study or by re-estimating the intercept using new patient data. Another important option is to extend the existing models by including new predictors, to both improve the discrimination performance and reduce heterogeneity in baseline risk. To address this, further work could include imputation of systematically missing predictors by borrowing information across studies; techniques for across-dataset imputation are only recently being developed [90–94], and further evidence on their performance is needed before implementation. There is a need to improve homogeneity across studies, for example in predictor measurement method, timing of predictor measurement, and outcome definition. The various risk thresholds that mothers would consider for making decisions on management need to be identified to apply the findings of decision curve analysis.

## Conclusion

A pre-eclampsia prediction model with good predictive performance would be beneficial to the UK NHS, but the evidence here suggests that, of the 24 models we could validate, their predictive performance is generally moderate, with miscalibration and heterogeneity across UK settings represented by the dataset available. Thus, there is not enough evidence to warrant recommendation for their routine use in clinical practice. Other models exist that we could not validate, which should also be examined in terms of their predictive performance, net benefit, and any heterogeneity across multiple UK settings before consideration for use in practice.

## Supplementary information

**Additional file 1: Supplementary methods:** Additional details for handling missing data and evaluating predictive performance of models. **Table S1:** Search strategy for pre-eclampsia prediction models. **Table S2:** Predictors evaluated in the models externally validated in the IPPIC-UK cohorts. **Table S3:** Prediction models and equations identified from the literature search. **Table S4:** Study level characteristics of IPPIC-UK cohorts. **Table S5:** Patient characteristics of IPPIC-UK cohorts. **Table S6:** Number and proportion missing for each predictor in each cohort used for external validation. **Table S7:** Risk of bias assessment of the IPPIC-UK cohorts using the PROBAST tool. **Table S8:** Summary of linear predictor values and predicted probabilities for each model in each cohort. **Table S9:** Predictive performance statistics for models in the individual IPPIC-UK cohorts. **Table S10:** Predictive performance statistics for models in nulliparous women in all cohorts and in the POP cohort. **Fig. S1:** Decision curves for early pre-eclampsia models in SCOPE, UPBEAT and POP. **Fig. S2:** Decision curves for late pre-eclampsia models in SCOPE, Allen 2017, UPBEAT and POP.

## Data Availability

The data that support the findings of this study are available from the IPPIC data sharing committee, but restrictions apply to the availability of these data, which were used under licence for the current study, and so are not publicly available. Data are however available from the authors upon reasonable request and with permission of contributing collaborators.

## Abbreviations

IPD: Individual participant data
IPPIC: International Prediction of Pregnancy Complications
BMI: Body mass index
MAP: Mean arterial pressure
PAPP-A: Pregnancy-associated plasma protein
LP: Linear predictor

## References

[1] Cantwell R, Clutton-Brock T, Cooper G, Dawson A, Drife J, Garrod D (2011). Saving mothers’ lives: reviewing maternal deaths to make motherhood safer: 2006-2008. The Eighth Report of the Confidential Enquiries into Maternal Deaths in the United Kingdom. BJOG.

[2] Ng VK, Lo TK, Tsang HH, Lau WL, Leung WC (2014). Intensive care unit admission of obstetric cases: a single centre experience with contemporary update. Hong Kong Med J.

[3] Kleinrouweler CE, Cheong-See Mrcog FM, Collins GS, Kwee A, Thangaratinam S, Khan KS, et al. Prognostic models in obstetrics: available, but far from applicable. Am J Obstet Gynecol. 2016;214(1):79–90.e36. 10.1016/j.ajog.2015.06.013 26070707

[4] Herraiz I, Arbues J, Camano I, Gomez-Montes E, Graneras A, Galindo A (2009). Application of a first-trimester prediction model for pre-eclampsia based on uterine arteries and maternal history in high-risk pregnancies. Prenat Diagn.

[5] Farina A, Rapacchia G, Freni Sterrantino A, Pula G, Morano D, Rizzo N (2011). Prospective evaluation of ultrasound and biochemical-based multivariable models for the prediction of late pre-eclampsia. Prenat Diagn.

[6] Meertens LJE, Scheepers HCJ, van Kuijk SMJ, Aardenburg R, van Dooren IMA, Langenveld J, et al. External validation and clinical usefulness of first trimester prediction models for the risk of preeclampsia: a prospective cohort study. Fetal Diagn Ther. 2019;45(6):381–93. 10.1159/000490385 PMC6604271 30021205

[7] Riley RD, Ensor J, Snell KI, Debray TP, Altman DG, Moons KG (2016). External validation of clinical prediction models using big datasets from e-health records or IPD meta-analysis: opportunities and challenges. BMJ.

[8] Debray TP, Riley RD, Rovers MM, Reitsma JB, Moons KG, Cochrane IPDM-aMg (2015). Individual participant data (IPD) meta-analyses of diagnostic and prognostic modeling studies: guidance on their use. PLoS Med.

[9] Debray TPA, Moons KGM, Ahmed I, Koffijberg H, Riley RD (2013). A framework for developing, implementing, and evaluating clinical prediction models in an individual participant data meta-analysis. Stat Med.

[10] Debray TP, Vergouwe Y, Koffijberg H, Nieboer D, Steyerberg EW, Moons KG (2015). A new framework to enhance the interpretation of external validation studies of clinical prediction models. J Clin Epidemiol.

[11] Lisonkova S, Joseph KS (2013). Incidence of preeclampsia: risk factors and outcomes associated with early- versus late-onset disease. Am J Obstet Gynecol.

[12] Townsend R, Khalil A, Premakumar Y, Allotey J, Snell KIE, Chan C, et al. Prediction of pre-eclampsia: review of reviews. Ultrasound Obstet Gynecol. 2019;54(1):16–27. 10.1002/uog.20117 30267475

[13] Allotey J, Snell KIE, Chan C, Hooper R, Dodds J, Rogozinska E, et al. External validation, update and development of prediction models for pre-eclampsiausing an Individual Participant Data (IPD) meta-analysis: the International Prediction of Pregnancy Complication Network (IPPIC pre-eclampsia) protocol. Diagn Progn Res. 2017;1:16. 10.1186/s41512-017-0016-z PMC6460674 31093545

[14] National Institute for Health and Care Excellence. Hypertension in pregnancy: diagnosis and management: NICE guidance (CG107); 2010. [updated 01/2011. Available from: https://www.nice.org.uk/guidance/cg107/chapter/1-guidance. 31498578

[15] Myatt L, Redman CW, Staff AC, et al. Strategy for standardization of preeclampsia research study design. Hypertension. 2014;63(6):1293–301. 10.1161/HYPERTENSIONAHA.113.02664 24688121

[16] Wolff RF, Moons KGM, Riley RD, Whiting PF, Westwood M, Collins GS (2019). PROBAST: a tool to assess the risk of bias and applicability of prediction model studies. Ann Intern Med.

[17] van Buuren S, Boshuizen HC, Knook DL (1999). Multiple imputation of missing blood pressure covariates in survival analysis. Stat Med.

[18] White IR, Royston P, Wood AM (2011). Multiple imputation using chained equations: issues and guidance for practice. Stat Med.

[19] Meng XL (1994). Multiple-imputaiton inferences with uncongenial sources of input. Stat Sci.

[20] Rubin DB (1987). Multiple imputation for nonresponse in surveys.

[21] Hosmer DW, Lemeshow S (2000). Assessing the fit of the model. Applied logistic regression.

[22] Hosmer DW, Lemeshow S (2000). Applied logistic regression.

[23] Marshall A, Altman DG, Holder RL, Royston P. Combining estimates of interest in prognostic modelling studies after multiple imputation: current practice and guidelines. BMC Med Res Methodol. 2009;9:57. 10.1186/1471-2288-9-57 PMC2727536 19638200

[24] Wood AM, Royston P, White IR (2015). The estimation and use of predictions for the assessment of model performance using large samples with multiply imputed data. Biom J.

[25] Snell KI, Hua H, Debray TP, Ensor J, Look MP, Moons KG (2016). Multivariate meta-analysis of individual participant data helped externally validate the performance and implementation of a prediction model. J Clin Epidemiol.

[26] Debray TP, Damen JA, Snell KI, Ensor J, Hooft L, Reitsma JB (2017). A guide to systematic review and meta-analysis of prediction model performance. BMJ.

[27] Hartung J, Knapp G (2001). A refined method for the meta-analysis of controlled clinical trials with binary outcome. Stat Med.

[28] Langan D, Higgins JPT, Jackson D, Bowden J, Veroniki AA, Kontopantelis E (2019). A comparison of heterogeneity variance estimators in simulated random-effects meta-analyses. Res Synth Methods.

[29] Higgins JPT, Thompson SG, Spiegelhalter DJ (2009). A re-evaluation of random-effects meta-analysis. J R Stat Soc Ser A Stat Soc.

[30] Vickers AJ, Elkin EB (2006). Decision curve analysis: a novel method for evaluating prediction models. Med Decis Mak.

[31] Vickers AJ, Van Calster B, Steyerberg EW (2016). Net benefit approaches to the evaluation of prediction models, molecular markers, and diagnostic tests. BMJ.

[32] Collins GS, Reitsma JB, Altman DG, Moons KGM; members of the TRIPOD group. Transparent Reporting of a Multivariable Prediction Model for Individual Prognosis or Diagnosis (TRIPOD): The TRIPOD Statement. Eur Urol. 2015;67(6):1142–51. 10.1016/j.eururo.2014.11.025 25572824

[33] Moons KG, Altman DG, Reitsma JB, Ioannidis JP, Macaskill P, Steyerberg EW (2015). Transparent Reporting of a multivariable prediction model for Individual Prognosis or Diagnosis (TRIPOD): explanation and elaboration. Ann Intern Med.

[34] Snell KI, Ensor J, Debray TP, Moons KG, Riley RD. Meta-analysis of prediction model performance across multiple studies: Which scale helps ensure between-study normality for the C-statistic and calibration measures?. Stat Methods Med Res. 2018;27(11):3505–22. 10.1177/0962280217705678 PMC6193210 28480827

[35] Wright D, Syngelaki A, Akolekar R, Poon LC, Nicolaides KH (2015). Competing risks model in screening for preeclampsia by maternal characteristics and medical history. Am J Obstet Gynecol.

[36] North RA, McCowan LM, Dekker GA, Poston L, Chan EH, Stewart AW (2011). Clinical risk prediction for pre-eclampsia in nulliparous women: development of model in international prospective cohort. BMJ.

[37] Allen RE, Zamora J, Arroyo-Manzano D, Velauthar L, Allotey J, Thangaratinam S (2017). External validation of preexisting first trimester preeclampsia prediction models. Eur J Obstet Gynecol Reprod Biol.

[38] Fraser A, Macdonald-Wallis C, Tilling K, Boyd A, Golding J, Davey Smith G (2013). Cohort profile: the Avon Longitudinal Study of Parents and Children: ALSPAC mothers cohort. Int J Epidemiol.

[39] Chappell LC, Seed PT, Briley AL, Kelly FJ, Lee R, Hunt BJ (1999). Effect of antioxidants on the occurrence of pre-eclampsia in women at increased risk: a randomised trial. Lancet.

[40] Chiswick C, Reynolds RM, Denison F, Drake AJ, Forbes S, Newby DE (2015). Effect of metformin on maternal and fetal outcomes in obese pregnant women (EMPOWaR): a randomised, double-blind, placebo-controlled trial. Lancet Diabetes Endocrinol.

[41] Poston L, Briley AL, Seed PT, Kelly FJ, Shennan AH (2006). Vitamin C and vitamin E in pregnant women at risk for pre-eclampsia (VIP trial): randomised placebo-controlled trial. Lancet.

[42] Poston L, Bell R, Croker H, Flynn AC, Godfrey KM, Goff L (2015). Effect of a behavioural intervention in obese pregnant women (the UPBEAT study): a multicentre, randomised controlled trial. Lancet Diabetes Endocrinol.

[43] Stirrup OT, Khalil A, D'Antonio F, Thilaganathan B, Southwest Thames Obstetric Research C (2015). Fetal growth reference ranges in twin pregnancy: analysis of the Southwest Thames Obstetric Research Collaborative (STORK) multiple pregnancy cohort. Ultrasound Obstet Gynecol.

[44] Ayorinde AA, Wilde K, Lemon J, Campbell D, Bhattacharya S (2016). Data resource profile: the Aberdeen Maternity and Neonatal Databank (AMND). Int J Epidemiol.

[45] Sovio U, White IR, Dacey A, Pasupathy D, Smith GCS (2015). Screening for fetal growth restriction with universal third trimester ultrasonography in nulliparous women in the Pregnancy Outcome Prediction (POP) study: a prospective cohort study. Lancet.

[46] Baschat AA, Magder LS, Doyle LE, Atlas RO, Jenkins CB, Blitzer MG (2014). Prediction of preeclampsia utilizing the first trimester screening examination. Am J Obstet Gynecol.

[47] Crovetto F, Figueras F, Triunfo S, Crispi F, Rodriguez-Sureda V, Dominguez C (2015). First trimester screening for early and late preeclampsia based on maternal characteristics, biophysical parameters, and angiogenic factors. Prenat Diagn.

[48] Goetzinger KR, Singla A, Gerkowicz S, Dicke JM, Gray DL, Odibo AO (2010). Predicting the risk of pre-eclampsia between 11 and 13 weeks’ gestation by combining maternal characteristics and serum analytes, PAPP-A and free beta-hCG. Prenat Diagn.

[49] Kuc S, Koster MP, Franx A, Schielen PC, Visser GH (2013). Maternal characteristics, mean arterial pressure and serum markers in early prediction of preeclampsia. PLoS One.

[50] Odibo AO, Zhong Y, Goetzinger KR, Odibo L, Bick JL, Bower CR (2011). First-trimester placental protein 13, PAPP-A, uterine artery Doppler and maternal characteristics in the prediction of pre-eclampsia. Placenta.

[51] Plasencia W, Maiz N, Bonino S, Kaihura C, Nicolaides KH (2007). Uterine artery Doppler at 11 + 0 to 13 + 6 weeks in the prediction of pre-eclampsia. Ultrasound Obstet Gynecol.

[52] Poon LC, Kametas NA, Chelemen T, Leal A, Nicolaides KH (2010). Maternal risk factors for hypertensive disorders in pregnancy: a multivariate approach. J Hum Hypertens.

[53] Poon LC, Kametas NA, Pandeva I, Valencia C, Nicolaides KH (2008). Mean arterial pressure at 11(+0) to 13(+6) weeks in the prediction of preeclampsia. Hypertension.

[54] Poon LC, Maiz N, Valencia C, Plasencia W, Nicolaides KH (2009). First-trimester maternal serum pregnancy-associated plasma protein-A and pre-eclampsia. Ultrasound Obstet Gynecol.

[55] Scazzocchio E, Figueras F, Crispi F, Meler E, Masoller N, Mula R (2013). Performance of a first-trimester screening of preeclampsia in a routine care low-risk setting. Am J Obstet Gynecol.

[56] Yu CK, Smith GC, Papageorghiou AT, Cacho AM, Nicolaides KH, Fetal Medicine Foundation Second Trimester Screening G (2005). An integrated model for the prediction of preeclampsia using maternal factors and uterine artery Doppler velocimetry in unselected low-risk women. Am J Obstet Gynecol.

[57] Boyd A, Golding J, Macleod J, Lawlor DA, Fraser A, Henderson J (2013). Cohort profile: the ‘children of the 90s’--the index offspring of the Avon Longitudinal Study of Parents and Children. Int J Epidemiol.

[58] Levine RJ, Lindheimer MD (2009). First-trimester prediction of early preeclampsia: a possibility at last!. Hypertension.

[59] Oliveira N, Magder LS, Blitzer MG, Baschat AA (2014). First-trimester prediction of pre-eclampsia: external validity of algorithms in a prospectively enrolled cohort. Ultrasound Obstet Gynecol.

[60] Park FJ, Leung CH, Poon LC, Williams PF, Rothwell SJ, Hyett JA (2013). Clinical evaluation of a first trimester algorithm predicting the risk of hypertensive disease of pregnancy. Aust N Z J Obstet Gynaecol.

[61] Skrastad RB, Hov GG, Blaas HG, Romundstad PR, Salvesen KA (2015). Risk assessment for preeclampsia in nulliparous women at 11-13 weeks gestational age: prospective evaluation of two algorithms. BJOG.

[62] Akolekar R, Syngelaki A, Poon L, Wright D, Nicolaides KH (2013). Competing risks model in early screening for preeclampsia by biophysical and biochemical markers. Fetal Diagn Ther.

[63] Gallo DM, Wright D, Casanova C, Campanero M, Nicolaides KH (2016). Competing risks model in screening for preeclampsia by maternal factors and biomarkers at 19–24 weeks’ gestation. Am J Obstet Gynecol.

[64] O'Gorman N, Wright D, Poon LC, Rolnik DL, Syngelaki A, de Alvarado M (2017). Multicenter screening for pre-eclampsia by maternal factors and biomarkers at 11-13 weeks’ gestation: comparison with NICE guidelines and ACOG recommendations. Ultrasound Obstet Gynecol.

[65] O'Gorman N, Wright D, Poon LC, Rolnik DL, Syngelaki A, Wright A (2017). Accuracy of competing-risks model in screening for pre-eclampsia by maternal factors and biomarkers at 11-13 weeks’ gestation. Ultrasound Obstet Gynecol.

[66] O'Gorman N, Wright D, Syngelaki A, Akolekar R, Wright A, Poon LC (2016). Competing risks model in screening for preeclampsia by maternal factors and biomarkers at 11–13 weeks gestation. Am J Obstet Gynecol.

[67] Poon LC, Kametas NA, Maiz N, Akolekar R, Nicolaides KH (2009). First-trimester prediction of hypertensive disorders in pregnancy. Hypertension.

[68] Poon LC, Syngelaki A, Akolekar R, Lai J, Nicolaides KH (2013). Combined screening for preeclampsia and small for gestational age at 11-13 weeks. Fetal Diagn Ther.

[69] Rolnik DL, Wright D, Poon LCY, Syngelaki A, O'Gorman N, de Paco MC (2017). ASPRE trial: performance of screening for preterm pre-eclampsia. Ultrasound Obstet Gynecol.

[70] Wright D, Akolekar R, Syngelaki A, Poon LC, Nicolaides KH (2012). A competing risks model in early screening for preeclampsia. Fetal Diagn Ther.

[71] Akolekar R, Syngelaki A, Sarquis R, Zvanca M, Nicolaides KH (2011). Prediction of early, intermediate and late pre-eclampsia from maternal factors, biophysical and biochemical markers at 11-13 weeks. Prenat Diagn.

[72] Akolekar R, Etchegaray A, Zhou Y, Maiz N, Nicolaides KH (2009). Maternal serum activin a at 11-13 weeks of gestation in hypertensive disorders of pregnancy. Fetal Diagn Ther.

[73] Akolekar R, Minekawa R, Veduta A, Romero XC, Nicolaides KH (2009). Maternal plasma inhibin A at 11-13 weeks of gestation in hypertensive disorders of pregnancy. Prenat Diagn.

[74] Akolekar R, Veduta A, Minekawa R, Chelemen T, Nicolaides KH (2011). Maternal plasma P-selectin at 11 to 13 weeks of gestation in hypertensive disorders of pregnancy. Hypertens Pregnancy.

[75] Akolekar R, Zaragoza E, Poon LC, Pepes S, Nicolaides KH (2008). Maternal serum placental growth factor at 11 + 0 to 13 + 6 weeks of gestation in the prediction of pre-eclampsia. Ultrasound Obstet Gynecol.

[76] Akolekar R, Syngelaki A, Beta J, Kocylowski R, Nicolaides KH (2009). Maternal serum placental protein 13 at 11-13 weeks of gestation in preeclampsia. Prenat Diagn.

[77] Garcia-Tizon Larroca S, Tayyar A, Poon LC, Wright D, Nicolaides KH (2014). Competing risks model in screening for preeclampsia by biophysical and biochemical markers at 30-33 weeks’ gestation. Fetal Diagn Ther.

[78] Tayyar A, Garcia-Tizon Larroca S, Poon LC, Wright D, Nicolaides KH (2014). Competing risk model in screening for preeclampsia by mean arterial pressure and uterine artery pulsatility index at 30-33 weeks' gestation. Fetal Diagn Ther.

[79] Lai J, Garcia-Tizon Larroca S, Peeva G, Poon LC, Wright D, Nicolaides KH (2014). Competing risks model in screening for preeclampsia by serum placental growth factor and soluble fms-like tyrosine kinase-1 at 30-33 weeks’ gestation. Fetal Diagn Ther.

[80] Riley RD, van der Windt D, Croft P, Moons KG (2019). Prognosis research in healthcare: concepts, methods and impact.

[81] Tan MY, Syngelaki A, Poon LC, Rolnik DL, O'Gorman N, Delgado JL (2018). Screening for pre-eclampsia by maternal factors and biomarkers at 11-13 weeks’ gestation. Ultrasound Obstet Gynecol.

[82] Rolnik DL, Wright D, Poon LC, O'Gorman N, Syngelaki A, de Paco MC (2017). Aspirin versus placebo in pregnancies at high risk for preterm preeclampsia. N Engl J Med.

[83] Wright D, Tan MY, O'Gorman N, Poon LC, Syngelaki A, Wright A (2019). Predictive performance of the competing risk model in screening for preeclampsia. Am J Obst Gynecol.

[84] Collins GS, de Groot JA, Dutton S, Omar O, Shanyinde M, Tajar A (2014). External validation of multivariable prediction models: a systematic review of methodological conduct and reporting. BMC Med Res Methodol.

[85] Le Cessie S, Van Houwelingen JC (1992). Ridge estimators in logistic regression. J R Stat Soc Ser C Appl Stat.

[86] Tibshirani R (1996). Regression shrinkage and selection via the lasso. J R Stat Soc Ser B Methodol.

[87] Zou H, Hastie T (2005). Regularization and variable selection via the elastic net. J R Stat Soc Ser Stat Methodol.

[88] Pavlou M, Ambler G, Seaman S, De Iorio M, Omar RZ (2016). Review and evaluation of penalised regression methods for risk prediction in low-dimensional data with few events. Stat Med.

[89] Steyerberg EW, Harrell FE, Borsboom GJJM, Eijkemans MJC, Vergouwe Y, Habbema JDF (2001). Internal validation of predictive models: efficiency of some procedures for logistic regression analysis. J Clin Epidemiol.

[90] Audigier V, White IR, Jolani S, Debray TPA, Quartagno M, Carpenter J (2018). Multiple imputation for multilevel data with continuous and binary variables. Stat Sci.

[91] Held U, Kessels A, Garcia Aymerich J, Basagana X, Ter Riet G, Moons KG (2016). Methods for handling missing variables in risk prediction models. Am J Epidemiol.

[92] Jolani S, Debray TP, Koffijberg H, van Buuren S, Moons KG (2015). Imputation of systematically missing predictors in an individual participant data meta-analysis: a generalized approach using MICE. Stat Med.

[93] Resche-Rigon M, White IR (2018). Multiple imputation by chained equations for systematically and sporadically missing multilevel data. Stat Methods Med Res.

[94] Quartagno M, Carpenter JR (2016). Multiple imputation for IPD meta-analysis: allowing for heterogeneity and studies with missing covariates. Stat Med.

