# Supplementary material for: External validation of prognostic models predicting pre-eclampsia: individual participant data meta-analysis
Source: BMC Med. 2020 Nov 2;18:302. doi: 10.1186/s12916-020-01766-9 (PMC7604970; doi:10.1186/s12916-020-01766-9)

Additional File 1

**Supplementary methods**

*Missing data checks*

Multiple imputation assumes that data are ‘missing at random’. While this is an untestable assumption, the following checks were performed to explore the missingness and whether ‘missing at random’ might be a reasonable assumption. Within each dataset, a missingness indicator variable was created (1=missing, 0=not missing) for each predictor with missing data. Logistic regression models were fitted to see if the missingness for each predictor could be predicted using the other predictors. T-tests were also performed to test for differences in continuous predictor values based on the missingness indicator for another variable.

No obvious missing data patterns were detected. Based on the preliminary checks, we found that the missingness of certain variables could be predicted by other variables and therefore we ensured that these other variables were included as auxiliary variables in the imputation models to improve the validity of the missing at random assumption.

*Evaluating predictive performance of models*

Each model was validated by first calculating the linear predictor (*LP*) of the model equation (*LP_i_*=*α* + *β*_1_*X_i_*_1_ + *β*_2_*X_i_*_2_ + …, where *α* is the intercept, *x* represents a predictor and *β* represents a predictor effect). For regression models (other than for time-to-event outcomes), the linear predictor is the right-hand side of the regression equation and is the linear combination of an intercept and the predictor values multiplied by their predictor effects. The linear predictor can then be transformed to get the predicted probability; for example, if the prediction model was developed using logistic regression, logit(*p_i_*) = *LP*_i_, so the predicted probability ($\text{p}$) of pre-eclampsia is calculated as $\text{p}_{\text{i}}\text{=}\frac{\text{e}^{\text{LP}_{\text{i}}}}{\text{1+}\text{e}^{\text{LP}_{\text{i}}}}$.

Discrimination is the ability of the model to separate between women who develop pre-eclampsia and those who do not. Calibration refers to how well the predicted risk from the model agrees with the observed risks for individuals in the dataset. The calibration slope is the slope of the regression line fitted to the relationship (across all individuals) between predicted and observed risk probabilities on the logit scale. The ideal slope value of 1 indicates overall agreement between observed and predicted risks across the whole range of predicted values; a slope <1 indicates overfitting where predictions are too extreme compared to observed probabilities (predictions are too high compared to what is observed for those at high risk and too low compared to what is observed for those at low risk), and slope >1 indicates underfitting where predictions are too narrow). The calibration-in-the-large (ideal value of 0) summarises whether the model over-predicts or under-predicts risk probabilities on average across all individuals. It indicates whether risk predictions are systematically too high (calibration-in-the-large <0) or too low (calibration-in-the-large >0).

Since the C-statistic is a proportion, it is unlikely to be normally distributed. Hence, we combined C-statistics across imputations on the logit scale (34) and standard errors for logit-C were calculated using the delta method as recommended (26). For the calibration plots, the predicted probability of pre-eclampsia for each individual was obtained by combining the imputation-specific estimates of the model’s linear predictor (using Rubin’s rules) and then applying the logit transformation (20).

Decision curves show the net benefit (i.e. benefit versus harm) over a range of clinically relevant threshold probabilities (i.e. for deciding to treat women when their predicted risk from the model is above a particular threshold value) and can be compared to the ‘treat all’ and ‘treat none’ strategies. For prediction models of binary outcomes, the net-benefit when multiplied by 1000 gives the additional number of true cases (those who will truly develop pre-eclampsia) identified for treatment, without increasing the number treated unnecessarily per 1000 individuals.

**Table S1: Search strategy for pre-eclampsia prediction models (MEDLINE via PubMed)**

| Set# | Searched for |
| --- | --- |
| #1 | Validat*[tiab] OR Predict*[ti] OR Rule*[tiab] |
| #2 | Predict*[tiab] AND (Outcome*[tiab] OR Risk*[tiab] OR Model*[tiab]) |
| #3 | (History[tiab] OR Variable*[tiab] OR Criteria[tiab] OR Scor*[tiab] OR Characteristic*[tiab] OR Finding*[tiab] OR Factor*[tiab]) AND (Predict*[tiab] OR Model*[tiab] OR Decision*[tiab] OR Identif*[tiab] OR Prognos*[tiab]) |
| #4 | Decision*[tiab] AND (Model*[tiab] OR Clinical*[tiab] OR Logistic Model*[tiab]) |
| #5 | Prognostic[tiab] AND (History[tiab] OR Variable*[tiab] OR Criteria[tiab] OR Scor*[tiab] OR Characteristic*[tiab] OR Finding*[tiab] OR Factor*[tiab] OR Model*[tiab]) |
| #6 | “risk score”[All fields] OR “prediction model”[All fields] OR “prediction rule”[All fields] OR “risk assessment”[All fields] OR “algorithm”[All fields] |
| #7 | # 1 OR #2 OR #3 OR #4 OR #5 OR #6 |
| #8 | (pregnan*[tiab] OR obstetric*[tiab] OR woman[tiab] OR women[tiab]) AND (preeclampsia[tiab] OR pre-eclampsia [tiab]) |
| #9 | #7 AND #8 |
| #10  #11 | #9 NOT (Animals[MeSH] NOT Humans[MeSH])  #10 Filters: from July 2012 – 2017 |

**Table S2: Predictors evaluated in the models externally validated in the IPPIC-UK cohorts.**

|  | **Model (as defined in Table 1)** | | | | | | | | | | | | | | | | | | | | | | | |
| --- | --- | --- | --- | --- | --- | --- | --- | --- | --- | --- | --- | --- | --- | --- | --- | --- | --- | --- | --- | --- | --- | --- | --- | --- |
| **Predictor** | **1: Plasencia 2007a** | **2: Poon 2008** | **3: Wright 2015a** | **4: Baschat 2014a** | **5: Goetzinger 2010** | **6: Odibo 2011a** | **7: Odibo 2011b** | **8: Yu 2005a** | **9: Baschat 2014b** | **10: Crovetto 2015a** | **11: Kuc 2013a** | **12: Plasencia 2007b** | **13: Poon 2010a** | **14: Scazzocchio 2013a** | **15: Wright 2015b** | **16: Poon 2009a** | **17: Yu 2005b** | **18: Crovetto 2015b** | **19: Kuc 2013b** | **20: Plasencia 2007c** | **21: Poon 2010b** | **22: Scazzocchio 2013b** | **23: Poon 2009b** | **24: Yu 2005c** |
| **Maternal clinical characteristics** |  |  |  |  |  |  |  |  |  |  |  |  |  |  |  |  |  |  |  |  |  |  |  |  |
| Ethnicity |  |  |  |  |  |  |  |  |  |  |  |  |  |  |  |  |  |  |  |  |  |  |  |  |
| BMI |  |  |  |  |  |  |  |  |  |  |  |  |  |  |  |  |  |  |  |  |  |  |  |  |
| History of PE in mother |  |  |  |  |  |  |  |  |  |  |  |  |  |  |  |  |  |  |  |  |  |  |  |  |
| Parity |  |  |  |  |  |  |  |  |  |  |  |  |  |  |  |  |  |  |  |  |  |  |  |  |
| Previous history of PE |  |  |  |  |  |  |  |  |  |  |  |  |  |  |  |  |  |  |  |  |  |  |  |  |
| Maternal age |  |  |  |  |  |  |  |  |  |  |  |  |  |  |  |  |  |  |  |  |  |  |  |  |
| Height |  |  |  |  |  |  |  |  |  |  |  |  |  |  |  |  |  |  |  |  |  |  |  |  |
| Chronic/pre-existing hypertension |  |  |  |  |  |  |  |  |  |  |  |  |  |  |  |  |  |  |  |  |  |  |  |  |
| Auto-immune disease |  |  |  |  |  |  |  |  |  |  |  |  |  |  |  |  |  |  |  |  |  |  |  |  |
| Mode of conception |  |  |  |  |  |  |  |  |  |  |  |  |  |  |  |  |  |  |  |  |  |  |  |  |
| Interval between pregnancies |  |  |  |  |  |  |  |  |  |  |  |  |  |  |  |  |  |  |  |  |  |  |  |  |
| Weight |  |  |  |  |  |  |  |  |  |  |  |  |  |  |  |  |  |  |  |  |  |  |  |  |
| Family history of PE |  |  |  |  |  |  |  |  |  |  |  |  |  |  |  |  |  |  |  |  |  |  |  |  |
| History of pre-existing diabetes |  |  |  |  |  |  |  |  |  |  |  |  |  |  |  |  |  |  |  |  |  |  |  |  |
| Mean Arterial Pressure |  |  |  |  |  |  |  |  |  |  |  |  |  |  |  |  |  |  |  |  |  |  |  |  |
| History of renal disease |  |  |  |  |  |  |  |  |  |  |  |  |  |  |  |  |  |  |  |  |  |  |  |  |
| Smoking |  |  |  |  |  |  |  |  |  |  |  |  |  |  |  |  |  |  |  |  |  |  |  |  |
| Previous heritable thrombophilia |  |  |  |  |  |  |  |  |  |  |  |  |  |  |  |  |  |  |  |  |  |  |  |  |
| Previous term live birth |  |  |  |  |  |  |  |  |  |  |  |  |  |  |  |  |  |  |  |  |  |  |  |  |
| **Ultrasound markers** |  |  |  |  |  |  |  |  |  |  |  |  |  |  |  |  |  |  |  |  |  |  |  |  |
| Uterine artery PI |  |  |  |  |  |  |  |  |  |  |  |  |  |  |  |  |  |  |  |  |  |  |  |  |
| Bilateral notching |  |  |  |  |  |  |  |  |  |  |  |  |  |  |  |  |  |  |  |  |  |  |  |  |
| **Biochemical markers** |  |  |  |  |  |  |  |  |  |  |  |  |  |  |  |  |  |  |  |  |  |  |  |  |
| PAPP-A |  |  |  |  |  |  |  |  |  |  |  |  |  |  |  |  |  |  |  |  |  |  |  |  |
| PAPP-A (MoM) |  |  |  |  |  |  |  |  |  |  |  |  |  |  |  |  |  |  |  |  |  |  |  |  |

BMI = Body Mass Index; PE = Pre-eclampsia; PI = Pulsatility index, PAPP-A = Pregnancy-associated plasma protein A; MoM = Multiple of the median

**Table S3: Prediction models and equations identified from the literature search.^#^**

| **Author, year** | **Predictor category** | **Trimester of measurement of variables** | **Equation(s)** | **Included in validation** | **If no, reason for exclusion** |
| --- | --- | --- | --- | --- | --- |
| **Any onset pre-eclampsia** | | | | | |
| Baschat, 2014 | Clinical and biochemical markers | 1^st^ trimester | **LP:** -8.72 + 0.157(if nulliparous) + 0.341(if history of hypertension) + 0.635(if history of prior PE) + 0.064(MAP) - 0.186(PAPP-A, Ln MoM) | Yes |  |
| Odibo, 2011 | Clinical and biochemical markers  Clinical and ultrasound markers | 1^st^ trimester | **LP1:** - 3.389 - 0.716(PAPP-A, MoM) + 0.05(BMI) + 0.319(if black ethnicity) + 1.57(if history of chronic hypertension)  **LP2:** - 3.895 - 0.593(Mean UtPI) +0.944(if pre-gestational diabetes) + 0.059(BMI) + 1.532(if history of chronic hypertension) | Yes  Yes |  |
| Goetzinger, 2010 | Clinical and biochemical markers | 1^st^ trimester | **LP:** - 3.25 +0.51(if PAPP-A <10^th^ cenile) + 0.93(if BMI >25) + 0.94(if chronic hypertension) + 0.97(if diabetes) + 0.61(if African American ethnicity) | Yes |  |
| Wright, 2015 | Clinical characteristics | 1^st^ trimester | **LP:** Mean gestational age at delivery with PE = 54.3637 - 0.0206886(age, years - 35, if age≥35) + 0.11711(height, cm - 164) - 2.6786(if Afro-Caribbean ethnicity) - 1.129(if South Asian ethnicity) - 7.2897(if chronic hypertension) - 3.0519(if systemic lupus erythematosus or antiphospholipid syndrome) - 1.6327(if conception by in vitro fertilization) - 8.1667(if parous with previous preeclampsia) + 0.0271988(if parous with previous PE, previous gestation in weeks - 24)^2^ - 4.335(if parous with no previous PE) -4.15137651(if parous with no previous PE, interval between pregnancies in years)^-1^ + 9.21473572(if parous with no previous PE, interval between pregnancies in years)^-0.5^ - 0.0694096(if no chronic hypertension, weight in kg – 69) - 1.7154(if no chronic hypertension and family history of PE) - 3.3899(if no chronic hypertension and diabetes mellitus type 1 or 2) | Yes |  |
| Poon, 2008 | Clinical characteristics | 1^st^ trimester | **LP:** - 6.311 + 1.299(if Afro-Caribbean ethnicity) + 0.092(BMI) + 0.855(if woman’s mother had PE) - 1.481(if parous without previous PE) + 0.933(if parous with previous PE) | Yes |  |
| Plasencia, 2007 | Clinical characteristics | 1^st^ trimester | **LP:** - 6.253 + 1.432( if Afro-Caribbean ethnicity) + 1.465(if mixed ethnicity) + 0.084(BMI) + 0.81(if patient’s mother had PE) - 1.539(if parous without previous PE) + 1.049(if parous with previous PE) | Yes |  |
| Yu, 2005 | Clinical and ultrasound markers | 2^nd^ trimester | **LP:** 1.8552 + 5.9228(mean UtPI)^-2^ - 14.4474(mean UtPI)^-1^ - 0.5478(if smoker) + 0.6719(bilateral notch) + 0.0372(age) + 0.4949(if black ethnicity) + 1.5033(if history of PE) - 1.2217(if previous term live birth) + 0.0367(BMI) | Yes |  |
| Odibo, 2011 | Clinical and biochemical markers  Clinical and biochemical and ultrasound markers | 1^st^ trimester | **LP2:** - 2.607 - 0.502(PP13, MoM) + 0.759(if pre-gestational diabetic) + 0.777(if black ethnicity) +1.268(if history of chronic hypertension)  **LP4:** - 1.308 - 0.574(PP13, MoM) - 0.502(PAPP-A, MoM) - 0.643(Mean UtPI) + 0.799(if pre-gestational diabetic) + 0.664(if black ethnicity) + 1.340(if history of chronic hypertension) | No  No | Predictor not available in IPPIC-UK dataset  Predictor not available in IPPIC-UK dataset |
| North, 2011 | Clinical characteristics  Clinical and ultrasound markers | 15 weeks’ gestation | **LP1:** - 6.8855 - 0.0393(age, years) + 0.0659(MAP) + 0.0483(BMI) + 0.6861(if family history of PE) + 0.6232(if family history CHD) - 0.3881(woman’s birth weight, kg) + 0.7129(if vaginal bleeding ≥5 days) - 0.8033(if one miscarriage ≤10 weeks, same partner) - 0.9070(if ≥12 months to conceive) - 0.3733(if high fruit intake at 15 weeks) - 0.508(if alcohol consumed in first trimester) - 0.063(no. of cigarettes/day at 15 weeks)  **LP2:** - 9.1113 + 0.0634(MAP) + 0.0485(BMI) +0.6539(if family history of PE) + 0.6093(if family history of CHD) - 0.3787(participant’s birth weight, kg) + 0.6493(if vaginal bleeding ≥5 days) + 0.5008(if months in sexual relationship ≤ 6 months) + 0.5084(if bilateral notches) + 2.0802(mean Ut RI) - 0.8248(one miscarriage ≤10 weeks, same partner) - 0.8983(≥12 months to conceive) - 0.4389(high fruit intake at 15 weeks) - 0.5573(if alcohol consumed in first trimester) | No  No | Predictor not available in IPPIC-UK dataset  Predictor not available in IPPIC-UK dataset |
| Seed, 2011 | Clinical characteristics | 2^nd^ trimester | **LP:** - 1.2422 + 0.4061(if chronic hypertension) + 0.5071(if DBP > 70) - 0.3846(if DBP > 90) + 0.8890(if SBP > 120) + 0.7040(if previous preeclampsia) - 0.4043(if on folates) + 0.8311(if mother is Indian, Bangladeshi, Pakistani, African,or Afro-Caribbean) | No | Predictor not available in IPPIC-UK dataset |
| Emonts, 2008 | Clinical characteristics | Post pregnancy | **LP:** - 3.72 + 0.030(age, years) - 0.50(parity) + 0.15(gestation) + 1.89(if HTA in patient’s mother) + 0.14(BMI) + 0.079 (SBP) - 0.13(DBP) | No | Predictor not available in IPPIC-UK dataset |
| Kenny, 2014 | Clinical and biochemical and ultrasound markers | 1^st^ trimester | **LP:** −12.200−0.655 high fruit intake+0.054 body mass index (BMI)+0.065 mean arterial blood pressure (MAP)+2.569 mean uterine artery resistance index (RI)−0.311 placental growth factor (PlGF) artery RI+1.232 cystatin C/PlGF | No | Predictor not available in IPPIC-UK dataset |
| Direkvand-Moghadam, 2013 | Clinical characteristics | 1^st^ trimester | **LP:** 0.74 – 1.016 prior infertility + 0.72 chronic hypertension +1.69 prior preeclampsia | No | Predictor not available in IPPIC-UK dataset |
| Teixeira, 2014 | Clinical and biochemical markers | 1^st^ trimester | **LP:** -5.723+0.870 (if chronic hypertension) + 1.428 (if diabetic) -0.787 (if multiparous) + 3.952 (if history of PE) + 0.039 * (maternal age) + 6.159 * (maternal weight MoM, log) + 0.027* (CRL) +(-0.483)* NT + 0.766 *(Free B-HCG MoM, log) | No | Predictor not available in IPPIC-UK dataset |
| **Early-onset pre-eclampsia** | | | | | |
| Crovetto, 2015 | Clinical characteristics | 1^st^ trimester | **LP1:** - 5.177+ 2.383(if black ethnicity) - 1.105(if nulliparous) + 3.543(if parous with previous PE) + 2.229(if chronic hypertension) + 2.201(if renal disease) | Yes |  |
| Baschat, 2014 | Clinical characteristics | 1^st^ trimester | **LP:** - 5.803 + 0.302(if history of diabetes) + 0.767(if history of hypertension) + 0.00948(MAP) | Yes |  |
| Scazzocchio, 2013 | Clinical characteristics | 1^st^ trimester | **LP1:** - 7.703 + 0.086(BMI) + 1.708(if chronic hypertension) + 4.033(if renal disease) + 1.931( if parous with previous PE) + 0.005(if parous with no previous PE) | Yes |  |
| Kuc, 2013 | Clinical characteristics | 1^st^ trimester | **LP:** - 6.790 – 0.119(maternal height, cm) + 4.8565(maternal weight, Ln) + 1.845(if nulliparous) + 0.086(maternal age, years) + 1.353(if smoker) | Yes |  |
| Poon, 2010 (b) | Clinical characteristics | 1^st^ trimester | **LP:** - 5.674 + 1.267(if black ethnicity) + 2.193(if history of chronic hypertension) -1.184(if parous without previous PE) + 1.362(if parous with previous PE) + 1.537(if conceived with ovulation induction) | Yes |  |
| Poon, 2009 (c) | Clinical and biochemical markers | 1^st^ trimester | **LP:** - 6.413 - 3.612 (PAPP-A, Ln MoM) + 1.803(if history of chronic hypertension) + 1.564(if black ethnicity) - 1.005(if parous without previous PE) + 1.491(if parous with previous PE) | Yes |  |
| Plasencia, 2007 | Clinical characteristics | 1^st^ trimester | **LP:** - 6.431 + 1.680(if Afro-Caribbean ethnicity) + 1.889(if mixed ethnicity) + 2.822(if parous with previous PE) | Yes |  |
| Yu, 2005 | Ultrasound markers | 2^nd^ trimester | **LP:** - 9.81223 + 2.10910(mean UtPI)^3^ - 1.79921(mean UtPI)^3^ + 1.059463(if bilateral notch) | Yes |  |
| Wright, 2015 | Clinical characteristics | 1^st^ trimester | **LP:** Mean gestational age at delivery with PE = 54.3637 - 0.0206886(age, years - 35, if age≥35) + 0.11711(height, cm - 164) - 2.6786(if Afro-Caribbean ethnicity) - 1.129(if South Asian ethnicity) - 7.2897(if chronic hypertension) - 3.0519(if systemic lupus erythematosus or antiphospholipid syndrome) - 1.6327(if conception by in vitro fertilization) - 8.1667(if parous with previous preeclampsia) + 0.0271988(if parous with previous PE, previous gestation in weeks - 24)^2^ - 4.335(if parous with no previous PE) -4.15137651(if parous with no previous PE, interval between pregnancies in years)^-1^ + 9.21473572(if parous with no previous PE, interval between pregnancies in years)^-0.5^ - 0.0694096(if no chronic hypertension, weight in kg – 69) - 1.7154(if no chronic hypertension and family history of PE) - 3.3899(if no chronic hypertension and diabetes mellitus type 1 or 2) | Yes |  |
| Crovetto, 2015 | Clinical and ultrasound markers  Clinical and biochemical and ultrasound markers | 1^st^ trimester | a-priori risk = exp(LP1)/(1+exp(LP1))  **LP2:** - 21.99 + 12.25(a-prioiri risk, log_10_) + 11.516(MAP, MoM) + 3.784(mean UtPI, MoM)  **LP3:** 21.515 + 12.884(a-priori risk, log_10_) + 11.219(MAP, MoM) + 3.325(mean UtPI, MoM) - 7.346(PlGF, log_10_) + 3.559(sFLT-1, log_10_) | No  No | Predictor not available in IPPIC-UK dataset  Predictor not available in IPPIC-UK dataset |
| Parra-cordero, 2013 | Clinical and biochemical and ultrasound markers | 1^st^ trimester | **LP:** - 6.942 + 0.074(BMI) + 1.878(if smoker) + 2.1116(lowest UtPI, Ln MoM) - 0.671(PlGF, Ln MoM) | No | Predictor not available in IPPIC-UK dataset |
| Scazzocchio, 2013 | Clinical and ultrasound markers | 1^st^ trimester | a-priori risk = exp(LP1)/(1+exp(LP1))  **LP2:** - 0.32 + 2.681(a priori risk, Ln) + 13.12(mean UtPI, Ln MoM) + 25.733(MAP, Ln MoM) | No | Predictor not available in IPPIC-UK dataset |
| Odibo, 2011 | Clinical and biochemical and ultrasound markers | 1^st^ trimester | **LP:** - 4.678 - 0.443(PP13, MoM) - 0.009(PAPP-A, MoM) + 0.347(Mean UtPI) + 3.059(if history of chronic hypertension) | No | Predictor not available in IPPIC-UK dataset |
| Seed, 2011 | Clinical characteristics | 2^nd^ trimester | **LP:** - 2.693 + 1.735(if SBP > 140) + 1.004(if on antihypertensive therapy) - 0.9790(if previous PE in most recent pregnancy) + 2.121(if previous PE with delivery < 34 weeks) + 1.285(if mother is Indian, Bangladeshi, Pakistani, African) | No | Predictor not available in IPPIC-UK dataset |
| Poon, 2010 (a) | Clinical and biochemical markers  Clinical and biochemical markers | 1^st^ trimester | **LP1:** 3.022 + 2.652(maternal factor-derived a-priori risk for early-PE, Ln) - 6.056(PlGF, Ln MoM) + 3.103(inhibin-A, Ln MoM) + 9.753(TNF-R1, Ln MoM)  **LP2:** 2.547 + 2.518(combined a-priori risk for early-PE, Ln) - 6.012(PlGF, Ln MoM) | No  No | Predictor not available in IPPIC-UK dataset  Predictor not available in IPPIC-UK dataset |
| Poon, 2009 (a) | Clinical and biochemical markers | 1^st^ trimester | **LP:** - 8.776 + 14.177(UtPI, Ln MoM) + 42.960(MAP, Ln MoM) - 2.249(PAPP-A, Ln MoM) - 3.529(PlGF, Ln MoM) + 0.120(BMI) - 1.472(if parous with no previous PE) | No | Predictor not available in IPPIC-UK dataset |
| Poon, 2009 (b) | Clinical and ultrasound markers | 1^st^ trimester | **LP:** - 3.657 + 1.592(maternal factor-derived *a-priori* risk for early PE, Ln) + 31.396(MAP, Ln MoM) + 13.322(lowest UtPI, Ln MoM) | No | Predictor not available in IPPIC-UK dataset |
| Akolekar, 2008 | Clinical and biochemical and ultrasound markers | 1^st^ trimester | **LP:** - 5.620 - 4.717(PlGF, Ln MoM) - 1.865(PAPP-A, Ln MoM) + 14.519(Ut PI, MoM) + 5.471(if history of chronic hypertension) + 1.159(if black ethnicity) | No | Predictor not available in IPPIC-UK dataset |
| Onwudiwe, 2008 | Clinical and ultrasound markers | 1^st^ and 2^nd^ trimester | **LP:** - 11.4487 + 31.2443(UtPI, Ln MoM) + 40.1105(MAP, Ln MoM) + 1.5442(if Afro-Caribbean ethnicity) | No | Predictor not available in IPPIC-UK dataset |
| Plasencia, 2008 | Clinical and ultrasound markers | 1^st^ trimester | **LP:** - 6.546 + 3.769(if chronic hypertension) + 15.692(UtPI, Ln MoM) | No | Predictor not available in IPPIC-UK dataset |
| Kenny, 2014 | Clinical and biochemical and ultrasound markers | 1^st^ trimester | **LP:** −14.164+0.075 MAP+6.1782 mean uterine artery RI+0.649 interleukin-1 receptor antagonist/PlGF; early-onset preeclampsia: −34.347+0.109 MAP+7.679 mean uterine | No | Predictor not available in IPPIC-UK dataset |
| Teixeira, 2014 | Clinical characteristics | 1^st^ trimester | **LP:** -4.951+1.519 (if chronic hypertension) -1.201 (if multiparous) + 3.201 (if history of PE) + 7.108 * (maternal weight MoM, log) | No | Predictor not available in IPPIC-UK dataset |
| Keikkala 2013 | Clinical and biochemical markers | 1^st^ trimester | **LP:** 1.75-3.27 * %hCG-h MoM -3.63 * PAPP-A  MoM +1.49 * parity (1 = nullipara, 0 = multipara) + 0.03 * MAP | No | Predictor not available in IPPIC-UK dataset |
| Meyers 2013 | Clinical characteristics  Clinical and biochemical markers  Clinical and ultrasound markers  Clinical and biochemical and ultrasound markers  Clinical and biochemical and ultrasound markers | 2^nd^ trimester | **LP1:** -8.4093 + 0.9037 9 fertility treatment + 0.7999 * any sister with pre-eclampsia + 0.1030 * MAP  **LP2:** -7.7769 + 0.7307 * fertility treatment + 0.1047 * MAP- 1.7269 * PlGF MOM  **LP3:** -13.5946 + 0.8402 * fertility treatment + 0.1039 * MAP + 7.0938 * 20 week mean uterine artery RI  **LP4:** -12.5382 + 0.1078 * MAP 1.5658 * PlGF MOM + 6.1087 * 20 week mean uterine artery RI  **LP5:** -10.4272 + 0.0994 * MAP 1.1787 * PlGF MOM + 0.0344 * endoglin 20 week + 0.5285 * 20 week bilateral notches of uterine arteries | No  No  No  No  No | Predictor not available in IPPIC-UK dataset  Predictor not available in IPPIC-UK dataset  Predictor not available in IPPIC-UK dataset  Predictor not available in IPPIC-UK dataset  Predictor not available in IPPIC-UK dataset |
| **Late-onset pre-eclampsia** | | | | | |
| Crovetto, 2015 | Clinical characteristics | 1^st^ trimester | **LP1:** - 5.873 - 0.462(if white ethnicity) + 0.109(BMI) - 0.825(if nulliparous) + 2.726(if parous with previous PE) + 1.956(if chronic hypertension) - 0.575(if smoker) | Yes |  |
| Scazzocchio, 2013 | Clinical characteristics | 1^st^ trimester | **LP1:** 6.135 + 2.124(if previous PE) + 1.571(if chronic hypertension) + 0.958(if diabetes) + 1.416(if thrombophilic condition) - 0.487(if multiparous) + 0.093(BMI) | Yes |  |
| Kuc, 2013 | Clinical characteristics | 1^st^ trimester | **LP:** - 14.374 + 2.300(maternal weight, Ln) + 1.303(if nulliparous) + 0.068(maternal age, years) | Yes |  |
| Poon, 2010 (b) | Clinical characteristics | 1^st^ trimester | **LP:** - 7.860 + 0.034(age, years) + 0.096(BMI) + 1.089(if black ethnicity) + 0.980(if Indian or Pakistani ethnicity) + 1.196(if mixed ethnicity) + 1.070(if woman’s mother had PE) - 1.413(if parous without previous PE) + 0.780(if parous with previous PE) | Yes |  |
| Poon, 2009 (c) | Clinical and biochemical markers | 1^st^ trimester | **LP:** - 6.652 - 0.884(PAPP-A, Ln MoM) + 1.127(if family history of PE) + 1.222(if black ethnicity) + 0.936(if Indian or Pakistani ethnicity) + 1.335(if mixed ethnicity) + 0.084(BMI) - 1.255(if parous without previous PE) + 0.818(if parous with previous PE) | Yes |  |
| Yu, 2005 | Clinical and ultrasound markers | 2^nd^ trimester | **LP:** 0.7901 + 5.1473(mean UtPI)^-2^ - 12.5152(mean UtPI)^-1^ - 0.5575(if smoker) + 0.5333(if bilateral notch) + 0.0328(age) + 0.4958(if black ethnicity) + 1.5109(if history of PE) + 1.1556(if previous term live birth) + 0.0378(BMI) | Yes |  |
| Plasencia, 2007 | Clinical characteristics | 1^st^ trimester | **LP:** Y = - 6.585 + 1.368(if Afro-Caribbean ethnicity) + 1.311(if mixed ethnicity) + 0.091(BMI) + 0.960(if patient’s mother had PE) - 1.663(if parous without previous PE) | Yes |  |
| Scazzocchio, 2013 | Clinical and biochemical markers | 1^st^ trimester | a-priori risk = exp(LP1)/(1+exp(LP1))  **LP2:** 0.328 + 2.205(a-priori risk, Ln) - 1.307(PAPP-A, Ln MoM) | No | Predictor not available in IPPIC-UK dataset |
| Crovetto, 2015 | Clinical and ultrasound markers  Clinical and biochemical and ultrasound markers | 1^st^ trimester | a-priori risk = exp(LP1)/(1+exp(LP1))  **LP2:** - 14.315+ 8.864(a-prioiri risk, log_10_) + 7.429(MAP, MoM) + 2.447(mean UtPI, MoM)  **LP3:** 25.921 + 9.652(a-priori risk, log_10_) + 6.89(MAP, MoM) + 2.343(mean UtPI, MoM) - 5.618(PlGF, log_10_) + 6.579(sFLT-1, log_10_) | No  No | Predictor not available in IPPIC-UK dataset  Predictor not available in IPPIC-UK dataset |
| Parra-cordero, 2013 | Clinical and biochemical and ultrasound markers | 1^st^ trimester | **LP:** - 5.584 + 0.137(BMI) + 0.822(lowest UtPI, Ln MoM) -0.533671(PlGF, Ln MoM) | No | Predictor not available in IPPIC-UK dataset |
| Poon, 2010 (a) | Clinical and biochemical markers  Clinical and biochemical markers | 1^st^ trimester | **LP1:** 3.810 + 2.898(maternal factor-derived a-priori risk for late-PE, Ln) - 3.171(PlGF, Ln MoM) + 3.792(activin-A, Ln MoM) + 2.013(MMP-9, Ln MoM) + 5.242(P-selectin, Ln MoM)  **LP2:** 3.490 + 2.717(combined a-priori risk for late-PE, Ln) - 2.966(PlGF, Ln MoM) + 3.937(activin-A, Ln MoM) + 4.190(P-selectin, Ln MoM) | No  No | Predictor not available in IPPIC-UK dataset  Predictor not available in IPPIC-UK dataset |
| Poon, 2009 (a) | Clinical and biochemical and ultrasound markers | 1^st^ trimester | **LP:** - 5.324 + 2.233(UtPI, Ln) + 23.134(MAP, Ln MoM) - 2.408(PlGF , Ln MoM) + 0.123(BMI) + 1.019(if black ethnicity) + 2.028(if mixed ethnicity) + 1.298(if family history of PE) - 1.443(if parous with no previous PE) | No | Predictor not available in IPPIC-UK dataset |
| Poon, 2009 (b) | Clinical and ultrasound markers | 1^st^ trimester | **LP:** - 0.468 + 2.272(maternal factor-derived *a-priori* risk for late PE, Ln) + 21.147(MAP, Ln MoM) + 3.537(lowest UtPI, Ln MoM) | No | Predictor not available in IPPIC-UK dataset |
| Akolekar, 2008 | Clinical and biochemical and ultrasound markers | 1^st^ trimester | **LP:** - 5.136 - 2.400(PlGF, Ln MoM) + 2.641(UtPI, Ln MoM) + 0.108(BMI) + 1.441(if patient’s mother had PE) + 1.366(if black ethnicity) + 1.083(if Indian or Pakistani ethnicity) + 1.549(if mixed ethnicity) - 1.281(if parous and no previous PE) | No | Predictor not available in IPPIC-UK dataset |
| Onwudiwe, 2008 | Clinical and ultrasound markers | 1^st^ and 2^nd^ trimester | **LP:** - 7.4924 + 6.2361(UtPI, Ln MoM) + 23.1953(MAP, Ln MoM) + 0.6003(if Afro-Caribbean ethnicity) + 0.1197(BMI) - 1.1058(if parous without previous PE) | No | Predictor not available in IPPIC-UK dataset |
| Plasencia, 2008 | Clinical and ultrasound markers | 1^st^ trimester | **LP:** - 6.140 + 0.082( BMI) + 0.813(if Afro-Caribbean ethnicity) - 1.234(if parous with no previous PE) + 0.922(if parous with a previous PE) + 1.049(if patient’s mother had PE) + 2.198(UtPI, Ln MoM) | No | Predictor not available in IPPIC-UK dataset |
| Kenny, 2014 | Clinical and biochemical and ultrasound markers | 1^st^ trimester | **LP:** −9.504−0.577 high fruit intake+0.058 MAP+0.058 BMI+0.550 tissue inhibitor of metalloproteinase 1 | No | Predictor not available in IPPIC-UK dataset |
| Teixeira, 2014 | Clinical and biochemical markers | 1^st^ trimester | **LP:** -8.248+0.050 (if chronic hypertension) + 1.649 (if diabetic) -0.623 (if multiparous) + 3.668 (if history of PE) + 5.834* (maternal weight MoM, log) + 0.036* (CRL) +(-0.592)* NT + 1.046 *(Free B-HCG MoM, log) | No | Predictor not available in IPPIC-UK dataset |

LP= linear predictor; PE=pre-eclampsia; BMI=body mass index; MAP=mean arterial pressure; CHD=coronary heart disease; SGA=small for gestational age; PAPP-A=pregnancy-associated plasma protein A; PlGF=placental growth factor; PP13= placental protein 13; UtA=uterine artery; PI=pulsatility index; RI=uterine resistance index. Ln and log_10_ indicate that a variable was modelled on the natural logarithm scale or logarithm with base 10 scale, respectively. linear predictor (*LP*) = *α* + *β*_1_**x*_1_ + *β*_2_**x*_2_ + …, and absolute predicted probabilities (*p*) can be obtained using the transformation $\text{p}\text{=}\frac{\text{e}^{\text{LP}}}{\text{1+}\text{e}^{\text{LP}}}$ ^.^

* For logistic regression, logit(*p*)=*LP* where the linear predictor (*LP*) = *α* + *β*_1_**x*_1_ + *β*_2_**x*_2_ + …, and absolute predicted probabilities (*p*) can be obtained using the transformation $\text{p}\text{=}\frac{\text{e}^{\text{LP}}}{\text{1+}\text{e}^{\text{LP}}}$ . The model for ‘mean gestational age at delivery with PE’ assumes a normal distribution with the predicted mean gestational age and SD=6.8833. The risk of delivery with PE is then calculated as the area under the normal curve between 24 weeks and either 42 weeks for any onset PE or 34 weeks for early-onset PE. For more detail see Wright et al., 2015 (35).

^#^Model equations were extracted from the original manuscripts, which may not incorporate later corrections to the equation by the authors if any

**Table S4: Study level characteristics of IPPIC-UK cohorts.**

| **Study/ Dataset** | **Study design** | **Data source** | **Data period** | **Population type** | **Inclusion criteria** | **Exclusion criteria** | **Contributing predictors** |
| --- | --- | --- | --- | --- | --- | --- | --- |
| SCOPE (36) | Observational | Prospective cohort | 2004-2008 | Low risk | Healthy nulliparous women with singleton pregnancies | Recognised as high risk of pre-eclampsia, small for gestational age baby or spontaneous preterm birth due to underlying medical condition such as chronic hypertension requiring antihypertensive drugs, diabetes, renal disease, systemic lupus erythematosus, antiphospholipid syndrome, sickle cell disease or HIV. Previous cervical knife cone biopsy, three or more abortions or miscarriages, current ruptured membranes, known major fetal anomaly or abnormal karyotype, and interventions that can alter the course of pregnancy such as aspirin or cervical suture. | Ethnicity BMI Family history of PE Parity and previous PE History of hypertension History of renal disease Spontaneous conception History of diabetes MAP Maternal height Maternal weight Nulliparous Maternal age Smoker Previous heritable thrombophilia  Bilateral notching |
| Allen, 2017 (37) | Observational | Prospective cohort | 2010-2014 | Any pregnancy | All pregnant women attending an inner London hospital | Women with multiple pregnancies and fetal anomalies | Ethnicity BMI Family history of PE Parity and previous PE History of hypertension History of renal disease Spontaneous conception History of diabetes MAP Maternal height Maternal weight Nulliparous Maternal age Smoker PAPP-A (MoM) PAPP-A Uterine PI |
| ALSPAC (38) | Observational | Prospective birth cohort | 1991-1992 | Any pregnancy | All pregnant women resident in Avon UK | None | Ethnicity  BMI  History of hypertension  History of renal disease  Spontaneous conception  History of diabetes  MAP  Maternal height  Maternal weight  Nulliparous  Maternal age  Smoker |
| Chappell, 1999 (39) | Randomised | Trial | NI | High risk | Pregnant women with an abnormal doppler waveform in either uterine artery at 18–22 weeks' gestation or a history of preeclampsia in a previous pregnancy which led to preterm delivery, eclampsia or HELLP syndrome | Heparin or warfarin treatment, abnormal fetal-anomaly scan or multiple pregnancy. | Ethnicity BMI Parity and previous PE History of hypertension History of diabetes Nulliparous Maternal age Smoker |
| EMPOWAR (40) | Randomised | Trial | 2011-2014 | High risk | Women at least 16 years of age at recruitment , between 12-16 weeks gestation and with a BMI of 30kg/m² | Non-white women and those with: history of diabetes, systemic disease at the time of enrolment (requiring either regular drugs or systemic corticosteroids treatment in the past 3 months), previous delivery of a baby smaller than the 3rd centile for weight, history of pre-eclampsia with delivery before 32 weeks’ gestation, known hypersensitivity to metformin hydrochloride or any of the excipients. Known liver or renal failure, acute disorders at the time of trial entry with the potential to change renal function, such as dehydration sufficient to require intravenous infusion, severe infection, shock, intravascular administration of iodinated contrast agents, or acute or chronic diseases that might cause tissue hypoxia (eg, cardiac or respiratory failure, recent myocardial infarction, hepatic insufficiency, acute alcohol intoxication, or alcoholism); lactating women; and women with multiple pregnancy | Ethnicity  BMI  Parity and previous PE  History of renal disease  History of diabetes  MAP  Maternal height  Maternal weight  Nulliparous  Maternal age  Smoker |
| Poston, 2006 (41) | Randomised | Trial | 2003-2005 | High risk | Gestational age 14-21 weeks plus one or more of the following risk factors: history of preeclampsia in preceding requiring preterm delivery, history of HELLP syndrome, eclampsia, essential hypertension requiring medication, maternal diastolic blood pressure of 90 mm Hg or more before 20 weeks’ gestation in the current pregnancy, history of diabetes, antiphospholipid syndrome;8 chronic renal disease, multiple pregnancy; abnormal uterine artery doppler waveform, primiparity with body-mass index (BMI) at first antenatal appointment of 30 kg/m² or more. | Women taking vitamin supplements containing doses of vitamin C of 200 mg or more or of vitamin E of 40 IU or more daily. Women treated with warfarin | BMI History of hypertension History of renal disease History of diabetes MAP Maternal height Maternal weight Maternal age  Bilateral notching |
| UPBEAT (42) | Randomised | Trial | 2009-2014 | High risk | Women older than 16 years with a BMI of 30 kg/m² or higher and a singleton pregnancy | Any underlying disorders, including a pre-pregnancy diagnosis of essential hypertension, diabetes, renal disease, systemic lupus erythematosus, antiphospholipid syndrome, sickle cell disease, thalassaemia, coeliac disease, thyroid disease, and current psychosis; or if on metformin. | Ethnicity  BMI  Family history of PE  Parity and previous PE  History of hypertension  History of renal disease  Spontaneous conception  History of diabetes  MAP  Maternal height  Maternal weight  Nulliparous  Maternal age  Smoker  PAPP-A |
| St Georges (43) | Observational | Prospective registry | 2000-2015 | Any pregnancy | All pregnant women attending an inner London hospital | None | Ethnicity BMI History of hypertension Spontaneous conception Maternal height Maternal weight Nulliparous Maternal age Smoker PAPP-A (MoM) Uterine PI  Bilateral notching |
| AMND (44) | Observational | Prospective registry | 1986-2015 | Any pregnancy | Data from every pregnancy event occurring in Aberdeen Maternity Hospital | None | BMI History of hypertension History of renal disease Maternal height Maternal weight Nulliparous Maternal age Smoker |
| Velauthar, 2012* | Observational | Prospective cohort | NI | Any pregnancy | All pregnant women attending an inner London hospital | None | Ethnicity BMI History of hypertension History of diabetes MAP Maternal height Maternal weight Nulliparous Smoker Uterine PI |
| POP (45) | Observational | Prospective cohort | 2008-2012 | Any pregnancy | Nulliparous women with singleton pregnancies | None | Ethnicity BMI Parity and previous PE History of hypertension History of renal disease Spontaneous conception History of diabetes MAP Maternal height Maternal weight Nulliparous Maternal age Smoker PAPP-A (MoM) PAPP-A Uterine PI  Bilateral notching |

PE= pre-eclampsia; BMI=Body mass index; MAP=Mean arterial pressure; PAPP-A=Pregnancy-associated plasma protein A; PI=Pulsatility index, NI=No information.

*Unpublished study

**Table S5: Patient characteristics of IPPIC-UK cohorts.**

| **Maternal Characteristics and outcomes** | **SCOPE (n=658)** | **Allen 2017**  **(n=1045)** | **ALSPAC (n=14344)** | **Chappell 1999**  **(n=316)** | **EMPOWAR (n=449)** | **Poston 2006**  **(n=2422)** | **UPBEAT**  **(n=1554)** | **St Georges (n=54635)** | **AMND (n=136635)** | **Velauthar 2012***  **(n=1145)** | | | **POP**  **(n=4212)** |
| --- | --- | --- | --- | --- | --- | --- | --- | --- | --- | --- | --- | --- | --- |
| Maternal age, mean (SD), range | 28.5 (5.6)  15 to 42 | 29.9 (5.1)  15 to 48 | 27.7 (4.9)  13 to 46 | 29.6 (5.9)  16 to 43 | 28.7 (5.4)  17 to 43 | 31.0 (5.8)  16 to 48 | 30.5 (5.5)  16 to 45 | 30.4 (5.6)  13 to 54 | 28.4 (5.6)  13 to 56 | | NR | 29.9 (5.1)  16 to 48 | |
| BMI, median [IQR], range | 24.1  [21.8, 26.8]  16.5 to 50.0 | 23.6  [21.1, 26.8]  14.8 to 51.1 | 21.5  [19.7, 23.7]  11.7 to 61.3 | 24.2  [21.7, 27.0]  16.4 to 56.5 | 38.2  [35.2, 41.8]  30.8 to 56.3 | 30.1  [24.6, 34.7]  16.6 to 65.4 | 35.1  [32.8, 38.5]  30.0 to 66.0 | 23.5  [21.3, 26.8]  11.9 to 79.8 | 28.0  [24.0, 32.0]  10.0 to 72.1 | | 23.9  [21.3, 27.3]  13.8 to 46.6 | 24.1 [21.8, 27.3]  14.7 to 54.7 | |
| Ethnicity, n (%)  White  Black  Asian  Hispanic  Mixed  Other | 554 (84)  49 (7)  47 (7)  1(<1)  0 (0)  7 (1) | 398 (38)  108 (10)  495 (47)  0 (0)  12 (1)  30 (3) | 11769 (97)  127 (1)  113 (<1)  0 (0)  0 (0)  76 (<1) | 215 (68)  91 (29)  6 (2)  3 (<1)  0 (0)  1 (<1) | 449 (100)  0 (0)  0 (0)  0 (0)  0 (0)  0 (0) | NR | 954 (61)  395 (25)  97 (6)  0 (0)  0 (0)  108 (7) | 33257 (62)  7820 (15)  10388 (19)  5 (<1)  1528 (3)  555 (1) | NR | | 402 (35)  90 (8)  565 (50)  0 (0)  0 (0)  82 (7) | 3900 (93)  25 (<1)  91 (2)  0 (0)  1 (<1)  195 (5) | |
| Nulliparous, n (%) | 658 (100) | 584 (56) | 5704 (45) | 202 (64) | 291 (65) | NR | 674 (43) | 29319 (54) | 65206 (48) | | 598 (52) | 4212 (100) | |
| Previous PE, n (%) | 0 (0) | 17 (2) | NR | 56 (18) | 0 (0) | 657 (27) | 69 (4) | NR | NR | | NR | 0 (0) | |
| Outcome, n (%)  Any PE  Early PE  Late PE | 32 (5)  6 (1)  26 (4) | 14 (1)  1 (<1)  13 (1) | 288 (2)  37 (<1)  251 (2) | 35 (12)  6 (2)  29 (10) | 10 (5)  0 (0)  10 (5) | 371 (15)  144 (6)  227 (9) | 54 (4)  5 (<1)  49 (3) | 1487 (3)  151 (<1)  1336 (2) | 4970 (4)  1237 (<1)  3733 (3) | | 26 (2)  3 (<1)  23 (2) | 273 (6)  10 (<1)  263 (6) | |

NR=not recorded; PE=pre-eclampsia. Note: summary of complete data only, therefore % is out of all those with recorded values rather than total individuals.

*Unpublished study

**Table S6: Number and proportion missing (or not recorded) for each predictor in each cohort used for external validation.**

| **Variable** | **N (%) missing** | | | | | | | | | | | |
| --- | --- | --- | --- | --- | --- | --- | --- | --- | --- | --- | --- | --- |
|  | **SCOPE (n=658)** | **Allen 2017**  **(n=1045)** | **ALSPAC (n=14344)** | **Chappell 1999**  **(n=316)** | **EMPOWAR**  **(n=449)** | **Poston 2006**  **(n=2422)** | **UPBEAT**  **(n=1554)** | **St Georges (n=54635)** | **AMND (n=136635)** | **Velauthar**  **2012**  **(n=1145)** | | **POP**  **(n=4212)** |
| **Predictors** |  |  |  |  |  |  |  |  |  | |  |  |
| Maternal age | 0 (0) | 1 (<1) | 1353 (9) | 0 (0) | 0 (0) | 22 (<1) | 0 (0) | 0 (0) | 19 (<1) | | 1145 (100) | 0 (0) |
| Ethnicity | 0 (0) | 2 (<1) | 2259 (16) | 0 (0) | 0 (0) | 2422 (100) | 0 (0) | 1082 (2) | 136635 (100) | | 6 (<1) | 0 (0) |
| Nulliparous | 0 (0) | 0 (0) | 1745 (12) | 0 (0) | 1 (<1) | 2422 (100) | 0 (0) | 104 (<1) | 6 (<1) | | 0 (0) | 0 (0) |
| Parity and previous PE | 0 (0) | 0 (0) | 14344 (100) | 0 (0) | 1 (<1) | 2422 (100) | 8 (<1) | 54635 (100) | 136635 (100) | | 1145 (100) | 0 (0) |
| Family history of PE | 0 (0) | 6 (<1) | 14344 (100) | 316 (100) | 449 (100) | 2422 (100) | 61 (4) | 54635 (100) | 136635 (100) | | 1145 (100) | 4212 (100) |
| Family history of PE in mother | 0 (0) | 6 (<1) | 14344 (100) | 316 (100) | 449 (100) | 2422 (100) | 101 (6) | 54635 (100) | 136635 (100) | | 1145 (100) | 4212 (100) |
| Spontaneous conception | 0 (0) | 0 (0) | 2167 (15) | 316 (100) | 449 (100) | 2422 (100) | 4 (<1) | 1427 (3) | 136635 (100) | | 1145 (100) | 0 (0) |
| History of hypertension | 0 (0) | 0 (0) | 2307 (16) | 0 (0) | 449 (100) | 0 (0) | 0 (0) | 0 (0) | 0 (0) | | 1 (<1) | 0 (0) |
| History of renal disease | 0 (0) | 0 (0) | 2060 (14) | 316 (100) | 0 (0) | 0 (0) | 0 (0) | 54635 (100) | 0 (0) | | 1145 (100) | 0 (0) |
| History of diabetes | 0 (0) | 0 (0) | 2119 (15) | 0 (0) | 0 (0) | 0 (0) | 0 (0) | 54635 (100) | 136635 (100) | | 1 (<1) | 0 (0) |
| Previous heritable thrombophilia | 0 (0) | 1045 (100) | 14344 (100) | 316 (100) | 449 (100) | 2422 (100) | 1554 (100) | 54635 (100) | 136635 (100) | | 1145 (100) | 4212 (100) |
| Smoker | 0 (0) | 0 (0) | 1972 (14) | 0 (0) | 0 (0) | 2422 (100) | 0 (0) | 4053 (7) | 2902 (2) | | 1 (<1) | 0 (0) |
| Maternal height | 0 (0) | 0 (0) | 8769 (61) | 316 (100) | 0 (0) | 0 (0) | 0 (0) | 8454 (15) | 3867 (3) | | 5 (<1) | 6 (<1) |
| Maternal weight (T1) | 0 (0) | 5 (<1) | 9190 (64) | 316 (100) | 152 (34) | 0 (0) | 0 (0) | 7992 (15) | 23926 (18) | | 6 (<1) | 146 (3) |
| T1 BMI | 0 (0) | 5 (<1) | 2409 (17) | 33 (10) | 152 (34) | 0 (0) | 0 (0) | 9151 (17) | 25650 (19) | | 6 (<1) | 152 (4) |
| T2 BMI | 0 (0) | 1040 (100) | 14344 (100) | 316 (100) | 297 (66) | 0 (0) | 0 (0) | 25183 (46) | 136635 (100) | | 1145 (100) | 57 (1) |
| T1 MAP | 0 (0) | 5 (<1) | 3618 (25) | 316 (100) | 152 (34) | 0 (0) | 1536 (99) | 54635 (100) | 136635 (100) | | 8 (<1) | 280 (7) |
| T1 PAPP-A (MoM) | 658 (100) | 119 (11) | 14344 (100) | 316 (100) | 449 (100) | 2422 (100) | 1554 (100) | 30919 (57) | 136635 (100) | | 1145 (100) | 171 (4) |
| T1 PAPP-A | 658 (100) | 119 (11) | 14344 (100) | 316 (100) | 449 (100) | 2422 (100) | 526 (34) | 54635 (100) | 136635 (100) | | 1145 (100) | 134 (3) |
| T1 UtPI | 658 (100) | 1045 (100) | 14344 (100) | 316 (100) | 449 (100) | 2422 (100) | 1554 (100) | 32595 (60) | 136635 (100) | | 2 (<1) | 4212 (100) |
| T2 UtPI | 658 (100) | 1045 (100) | 14344 (100) | 316 (100) | 449 (100) | 2257 (93) | 1554 (100) | 28109 (51) | 136635 (100) | | 1145 (100) | 133 (3) |
| T2 Binotch | 3 (<1) | 1040 (100) | 14344 (100) | 316 (100) | 449 (100) | 27 (1) | 1554 (100) | 21596 (40) | 136635 (100) | | 1145 (100) | 133 (3) |
| **Outcomes** |  |  |  |  |  |  |  |  |  | |  |  |
| Any PE | 4 (<1) | 0 (0) | 832 (5) | 33 (10) | 246 (55) | 0 (0) | 47 (3) | 0 (0) | 0 (0) | | 32 (3) | 5 (<1) |
| Early PE | 4 (<1) | 0 (0) | 832 (5) | 33 (10) | 78 (17) | 0 (0) | 47 (3) | 0 (0) | 0 (0) | | 32 (3) | 5 (<1) |
| Late PE | 4 (<1) | 0 (0) | 832 (5) | 33 (10) | 239 (53) | 0 (0) | 47 (3) | 0 (0) | 0 (0) | | 32 (3) | 5 (<1) |

PE= pre-eclampsia; BMI=Body mass index; MAP=Mean arterial pressure; PAPP-A=Pregnancy-associated plasma protein A; UtPI=Uterine artery pulsatility index

**Table S7: Risk of bias assessment of the IPPIC-UK cohorts using the PROBAST tool.**

| **Study/Dataset** | | **Domain: Participant Selection** | | | | | | | | | | | | | | |  |
| --- | --- | --- | --- | --- | --- | --- | --- | --- | --- | --- | --- | --- | --- | --- | --- | --- | --- |
|  |  | **Appropriate data sources** | | **Appropriate inclusion and exclusion of participants** | | | | **Participant selection similar to model development study** | | | | | **Risk of bias** | **Rationale of rating** | | |  |
| SCOPE | | Yes | | Yes | | | | N/A | | | | | Low | Responses to relevant signalling questions are yes | | |  |
| Allen 2017 | | Yes | | Yes | | | | N/A | | | | | Low | Responses to relevant signalling questions are yes | | |  |
| ALSPAC | | Yes | | Yes | | | | N/A | | | | | Low | Responses to relevant signalling questions are yes | | |  |
| Chappell 1999 | | Yes | | No | | | | N/A | | | | | High | Selected high-risk population | | |  |
| EMPOWAR | | Yes | | No | | | | N/A | | | | | High | Selected high-risk population | | |  |
| Poston 2006 | | Yes | | No | | | | N/A | | | | | High | Selected high-risk population | | |  |
| UPBEAT | | Yes | | No | | | | N/A | | | | | High | Selected high-risk population | | |  |
| St Georges | | Yes | | Yes | | | | N/A | | | | | Low | Responses to relevant signalling questions are yes | | |  |
| AMND | | Yes | | Yes | | | | N/A | | | | | Low | Responses to relevant signalling questions are yes | | |  |
| Velauthar 2012 | | Yes | | Yes | | | | N/A | | | | | Low | Responses to relevant signalling questions are yes | | |  |
| POP | | Yes | | Yes | | | | N/A | | | | | Low | Responses to relevant signalling questions are yes | | |  |
| **Study/Dataset** | | **Domain: Predictors** | | | | | | | | | | | | | | |  |
|  |  | **Predictors defined in a similar way for participants** | | **Predictors defined in a similar way to model development study** | | | **Predictors assessed without knowledge of outcome data** | | | **All predictors available at the time model is to be used** | | | **Risk of bias** | **Rationale of rating** | | |  |
| SCOPE | | Yes | | N/A | | | Yes | | | Yes | | | Low | Responses to relevant signalling questions are yes | | |  |
| Allen 2017 | | Yes | | N/A | | | Yes | | | Yes | | | Low | Responses to relevant signalling questions are yes | | |  |
| ALSPAC | | Yes | | N/A | | | Yes | | | Yes | | | Low | Responses to relevant signalling questions are yes | | |  |
| Chappell 1999 | | Yes | | N/A | | | Yes | | | Yes | | | Low | Responses to relevant signalling questions are yes | | |  |
| EMPOWAR | | Yes | | N/A | | | Yes | | | Yes | | | Low | Responses to relevant signalling questions are yes | | |  |
| Poston 2006 | | Yes | | N/A | | | Yes | | | Yes | | | Low | Responses to relevant signalling questions are yes | | |  |
| UPBEAT | | Yes | | N/A | | | Yes | | | Yes | | | Low | Responses to relevant signalling questions are yes | | |  |
| St Georges | | Probably yes | | N/A | | | Probably yes | | | Yes | | | Low | Responses to relevant signalling questions are yes | | |  |
| AMND | | Probably yes | | N/A | | | Probably yes | | | Yes | | | Low | Responses to relevant signalling questions are yes | | |  |
| Velauthar 2012 | | NI | | N/A | | | NI | | | Yes | | | Unclear | No information to make assessment | | |  |
| POP | | Yes | | N/A | | | Yes | | | Yes | | | Low | Responses to relevant signalling questions are yes | | |  |
| **Study/Dataset** | **Domain: Outcome** | | | | | | | | | | | | | | | | |
|  | **Outcome appropriately determined** | | **Pre-specified or standard definition used** | | **Predictors excluded from outcome definition** | **Outcome defined and determined in the same way for all participants** | | | **Outcome defined and determined in the same way to model development study** | | **Outcome determined without knowledge of predictor information** | **Time interval between predictor and outcome assessment appropriate** | | | **Risk of bias** | **Rationale of rating** | |
| SCOPE | Yes | | Yes | | No | Yes | | | N/A | | No | Yes | | | Low | Blood pressure and proteinuria are components of predictor and need to be known for diagnosis | |
| Allen 2017 | NI | | NI | | Probably no | Probably yes | | | N/A | | Probably no | Probably yes | | | Unclear | No information to make assessment | |
| ALSPAC | NI | | NI | | Probably no | Probably yes | | | N/A | | Probably no | Probably yes | | | Unclear | No information to make assessment | |
| Chappell 1999 | Yes | | Yes | | No | Yes | | | N/A | | No | Yes | | | Low | Blood pressure and proteinuria are components of predictor and need to be known for diagnosis | |
| EMPOWAR | NI | | NI | | Probably no | Probably yes | | | N/A | | Probably no | Probably yes | | | Unclear | No information to make assessment | |
| Poston 2006 | Yes | | Yes | | No | Yes | | | N/A | | No | Yes | | | Low | Blood pressure and proteinuria are components of predictor and need to be known for diagnosis | |
| UPBEAT | Yes | | Yes | | No | Yes | | | N/A | | No | Yes | | | Low | Blood pressure and proteinuria are components of predictor and need to be known for diagnosis | |
| St Georges | NI | | NI | | Probably no | Probably yes | | | N/A | | Probably no | Probably yes | | | Unclear | No information to make assessment | |
| AMND | NI | | NI | | Probably no | Probably yes | | | N/A | | Probably no | Probably yes | | | Unclear | No information to make assessment | |
| Velauthar 2012 | NI | | NI | | Probably no | Probably yes | | | N/A | | Probably no | Probably yes | | | Unclear | No information to make assessment | |
| POP | Yes | | Yes | | No | Yes | | | N/A | | No | Yes | | | Low | Blood pressure and proteinuria are components of predictor and need to be known for diagnosis | |

NI = no information; N/A= not applicable

**Table S8: Summary of linear predictor values and predicted probabilities for each model in each cohort.**

| **Model no.** | **First author (year)** | **Type of predictors** | **Study** | **N Total** | **No. Events* (%)** | **Linear predictor*** | | | **Predicted probability*** | | |
| --- | --- | --- | --- | --- | --- | --- | --- | --- | --- | --- | --- |
|  |  |  |  |  |  | **Median** | **Interquartile range** | **Range**  **(min to max)** | **Median** | **Interquartile range** | **Range**  **(min to max)** |
| **Trimester 1 any-onset pre-eclampsia models** | | | |  |  |  |  |  |  |  |  |
| 1 | Plasencia 2007a | C | SCOPE | 658 | 33 (5.0) | -4.186 | -4.408 to -3.868 | -4.865 to -1.052 | 0.015 | 0.012 to 0.020 | 0.008 to 0.259 |
|  |  |  | Allen 2017 | 1045 | 14 (1.3) | -4.508 | -5.576 to -4.139 | -6.464 to -1.023 | 0.011 | 0.004 to 0.016 | 0.002 to 0.264 |
|  |  |  | UPBEAT | 1554 | 56 (3.6) | -3.488 | -4.531 to -2.943 | -5.269 to 0.337 | 0.03 | 0.011 to 0.050 | 0.005 to 0.584 |
| 2 | Poon 2008 | C | SCOPE | 658 | 33 (5.0) | -4.047 | -4.290 to -3.700 | -4.790 to -0.884 | 0.017 | 0.014 to 0.024 | 0.008 to 0.292 |
|  |  |  | Allen 2017 | 1045 | 14 (1.3) | -4.406 | -5.386 to -4.002 | -6.337 to -0.885 | 0.012 | 0.005 to 0.018 | 0.002 to 0.292 |
|  |  |  | UPBEAT | 1554 | 56 (3.6) | -3.292 | -4.220 to -2.700 | -5.028 to 0.691 | 0.036 | 0.014 to 0.063 | 0.007 to 0.666 |
| 3 | Wright 2015a | C | SCOPE | 658 | 33 (5.0) | -3.272 | -3.513 to -2.929 | -4.179 to -0.758 | 0.037 | 0.029 to 0.051 | 0.015 to 0.319 |
|  |  |  | Allen 2017 | 584 | 8 (1.4) | -3.093 | -3.436 to -2.729 | -4.182 to -0.962 | 0.043 | 0.031 to 0.061 | 0.015 to 0.277 |
|  |  |  | UPBEAT | 674 | 36 (5.3) | -2.084 | -2.300 to -1.710 | -3.090 to -0.437 | 0.111 | 0.091 to 0.153 | 0.044 to 0.393 |
| 4 | Baschat 2014a | C+B | Allen 2017 | 1045 | 14 (1.3) | -3.041 | -3.440 to -2.615 | -4.705 to 0.283 | 0.046 | 0.031 to 0.068 | 0.009 to 0.570 |
|  |  |  | POP | 4212 | 273 (6.5) | -3.549 | -3.885 to -3.107 | -5.801 to 0.185 | 0.028 | 0.020 to 0.043 | 0.003 to 0.546 |
| 5 | Goetzinger 2010 | C+B | Allen 2017 | 1045 | 14 (1.3) | -3.25 | -3.250 to -2.320 | -3.250 to -0.260 | 0.037 | 0.037 to 0.089 | 0.037 to 0.435 |
|  |  |  | UPBEAT | 1554 | 56 (3.6) | -2.32 | -2.320 to -1.710 | -2.320 to -1.200 | 0.089 | 0.089 to 0.153 | 0.089 to 0.231 |
|  |  |  | POP | 4212 | 273 (6.5) | -3.25 | -3.250 to -2.320 | -3.250 to 0.100 | 0.037 | 0.037 to 0.089 | 0.037 to 0.525 |
| 6 | Odibo 2011a | C+B | Allen 2017 | 1045 | 14 (1.3) | -2.92 | -3.223 to -2.614 | -5.367 to -0.301 | 0.051 | 0.038 to 0.068 | 0.005 to 0.425 |
|  |  |  | St Georges | 54635 | 1487 (2.7) | -2.866 | -3.200 to -2.573 | -36.527 to 0.882 | 0.054 | 0.039 to 0.071 | 0.000 to 0.707 |
|  |  |  | POP | 4212 | 273 (6.5) | -2.874 | -3.287 to -2.517 | -21.418 to 0.230 | 0.053 | 0.036 to 0.075 | 0.000 to 0.557 |
| 7 | Odibo 2011b | C+U | Velauthar 2012 | 1145 | 28 (2.4) | -3.331 | -3.543 to -3.080 | -4.586 to -0.194 | 0.035 | 0.028 to 0.044 | 0.010 to 0.452 |
| **Trimester 2 any-onset pre-eclampsia models** | | | |  |  |  |  |  |  |  |  |
| 8 | Yu 2005a | C+U | POP | 4212 | 273 (6.5) | -4.470 | -4.806 to -3.860 | -6.206 to 65.058 | 0.011 | 0.008 to 0.021 | 0.002 to 1.000 |
| **Trimester 1 early-onset pre-eclampsia models** | | | |  |  |  |  |  |  |  |  |
| 9 | Baschat 2014b | C | SCOPE | 658 | 6 (0.9) | -5.070 | -5.114 to -5.019 | -5.234 to -4.069 | 0.006 | 0.006 to 0.007 | 0.005 to 0.017 |
|  |  |  | ALSPAC | 14344 | 40 (0.3) | -5.013 | -5.076 to -4.949 | -5.362 to -3.502 | 0.007 | 0.006 to 0.007 | 0.005 to 0.029 |
|  |  |  | Poston 2006 | 2422 | 144 (6.0) | -4.891 | -5.013 to -4.151 | -5.297 to -3.631 | 0.007 | 0.007 to 0.016 | 0.005 to 0.026 |
|  |  |  | Velauthar 2012 | 1145 | 4 (0.3) | -5.029 | -5.090 to -4.975 | -5.272 to -3.867 | 0.007 | 0.006 to 0.007 | 0.005 to 0.020 |
|  |  |  | POP | 4212 | 10 (0.2) | -5.059 | -5.108 to -4.992 | -5.361 to -3.761 | 0.006 | 0.006 to 0.007 | 0.005 to 0.023 |
| 10 | Crovetto 2015a | C | SCOPE | 658 | 6 (0.9) | -6.282 | -6.282 to -6.282 | -6.282 to -1.670 | 0.002 | 0.002 to 0.002 | 0.002 to 0.158 |
|  |  |  | UPBEAT | 1554 | 6 (0.4) | -5.177 | -6.282 to -3.899 | -6.282 to 0.749 | 0.006 | 0.002 to 0.020 | 0.002 to 0.679 |
|  |  |  | POP | 4212 | 10 (0.2) | -6.282 | -6.282 to -6.282 | -6.282 to -1.670 | 0.002 | 0.002 to 0.002 | 0.002 to 0.158 |
| 11 | Kuc 2013a | C | SCOPE | 658 | 6 (0.9) | -1.679 | -2.24 to -0.955 | -4.285 to 2.445 | 0.157 | 0.096 to 0.278 | 0.014 to 0.920 |
|  |  |  | ALSPAC | 14344 | 40 (0.3) | -3.339 | -4.242 to -2.413 | -8.414 to 2.483 | 0.034 | 0.014 to 0.082 | 0.000 to 0.919 |
|  |  |  | UPBEAT | 1554 | 6 (0.4) | -0.543 | -1.413 to 0.373 | -3.744 to 3.982 | 0.367 | 0.196 to 0.592 | 0.023 to 0.982 |
|  |  |  | St Georges | 54635 | 151 (0.3) | -2.351 | -3.236 to -1.488 | -11.455 to 7.092 | 0.087 | 0.038 to 0.184 | 0.000 to 0.999 |
|  |  |  | AMND | 136635 | 1237 (0.9) | -2.304 | -3.223 to -1.371 | -7.660 to 4.857 | 0.091 | 0.038 to 0.202 | 0.000 to 0.992 |
|  |  |  | POP | 4212 | 10 (0.2) | -1.599 | -2.268 to -0.842 | -4.585 to 3.497 | 0.168 | 0.094 to 0.301 | 0.010 to 0.970 |
| 12 | Plasencia 2007b | C | SCOPE | 658 | 6 (0.9) | -6.431 | -6.431 to -6.431 | -6.431 to -4.751 | 0.002 | 0.002 to 0.002 | 0.002 to 0.009 |
|  |  |  | Chappell 1999 | 316 | 7 (2.1) | -6.431 | -6.431 to -4.751 | -6.431 to -1.929 | 0.002 | 0.002 to 0.009 | 0.002 to 0.127 |
|  |  |  | UPBEAT | 1554 | 6 (0.4) | -6.431 | -6.431 to -4.751 | -6.431 to -1.929 | 0.002 | 0.002 to 0.009 | 0.002 to 0.127 |
|  |  |  | POP | 4212 | 10 (0.2) | -6.431 | -6.431 to -6.431 | -6.431 to -4.542 | 0.002 | 0.002 to 0.002 | 0.002 to 0.011 |
| 13 | Poon 2010a | C | SCOPE | 658 | 6 (0.9) | -5.674 | -5.674 to -5.674 | -5.674 to -2.214 | 0.003 | 0.003 to 0.003 | 0.003 to 0.099 |
|  |  |  | UPBEAT | 1554 | 6 (0.4) | -4.137 | -5.321 to -4.054 | -6.858 to -1.508 | 0.016 | 0.005 to 0.017 | 0.001 to 0.181 |
|  |  |  | POP | 4212 | 10 (0.2) | -4.137 | -4.137 to -4.137 | -5.674 to -0.677 | 0.016 | 0.016 to 0.016 | 0.003 to 0.337 |
| 14 | Scazzocchio 2013a | C | SCOPE | 658 | 6 (0.9) | -5.631 | -5.832 to -5.397 | -6.282 to -3.145 | 0.004 | 0.003 to 0.005 | 0.002 to 0.041 |
|  |  |  | UPBEAT | 1554 | 6 (0.4) | -4.662 | -4.877 to -4.333 | -5.127 to -0.099 | 0.009 | 0.008 to 0.013 | 0.006 to 0.475 |
|  |  |  | POP | 4212 | 10 (0.2) | -5.617 | -5.822 to -5.303 | -6.448 to 1.688 | 0.004 | 0.003 to 0.005 | 0.002 to 0.844 |
| 15 | Wright 2015b | C | SCOPE | 658 | 6 (0.9) | -6.461 | -6.804 to -5.972 | -7.744 to -2.932 | 0.002 | 0.001 to 0.003 | 0.000 to 0.051 |
|  |  |  | UPBEAT | 674 | 6 (0.4) | -4.761 | -5.069 to -4.231 | -6.201 to -2.521 | 0.008 | 0.006 to 0.014 | 0.002 to 0.074 |
| 16 | Poon 2009a | C+B | POP | 4212 | 10 (0.2) | -6.321 | -7.824 to -4.693 | -18.294 to 3.971 | 0.002 | 0.000 to 0.009 | 0.000 to 0.981 |
| **Trimester 2 early-onset pre-eclampsia models** | | | |  |  |  |  |  |  |  |  |
| 17 | Yu 2005b | C+U | POP | 4212 | 10 (0.2) | -9.601 | -9.694 to -9.412 | -9.809 to 0.374 | 0.000 | 0.000 to 0.000 | 0.000 to 0.591 |
| **Trimester 1 late-onset pre-eclampsia models** | | | |  |  |  |  |  |  |  |  |
| 18 | Crovetto 2015b | C | SCOPE | 658 | 26 (4.0) | -4.504 | -4.789 to -4.149 | -5.703 to -1.592 | 0.011 | 0.008 to 0.016 | 0.003 to 0.169 |
|  |  |  | Allen 2017 | 1045 | 13 (1.2) | -3.914 | -4.494 to -3.298 | -5.654 to 1.509 | 0.020 | 0.011 to 0.036 | 0.003 to 0.819 |
|  |  |  | Chappell 1999 | 316 | 32 (10.0) | -4.084 | -4.648 to -2.645 | -5.903 to 2.098 | 0.017 | 0.009 to 0.066 | 0.003 to 0.891 |
|  |  |  | UPBEAT | 1554 | 51 (3.3) | -2.658 | -3.207 to -2.114 | -4.468 to 3.007 | 0.066 | 0.039 to 0.108 | 0.011 to 0.953 |
|  |  |  | POP | 4212 | 263 (6.2) | -4.496 | -4.775 to -4.109 | -6.133 to -0.074 | 0.011 | 0.008 to 0.016 | 0.002 to 0.481 |
| 19 | Kuc 2013b | C | SCOPE | 658 | 26 (4.0) | -1.464 | -1.871 to -1.062 | -3.016 to 0.191 | 0.188 | 0.133 to 0.257 | 0.047 to 0.548 |
|  |  |  | Allen 2017 | 1045 | 13 (1.2) | -2.104 | -2.653 to -1.526 | -4.520 to 0.558 | 0.109 | 0.066 to 0.179 | 0.011 to 0.636 |
|  |  |  | ALSPAC | 14344 | 266 (1.9) | -2.698 | -3.226 to -2.070 | -5.045 to 0.134 | 0.063 | 0.038 to 0.112 | 0.006 to 0.533 |
|  |  |  | EMPOWAR | 449 | 28 (6.3) | -0.801 | -1.395 to -0.281 | -3.032 to 1.118 | 0.310 | 0.199 to 0.430 | 0.047 to 0.751 |
|  |  |  | UPBEAT | 1554 | 51 (3.3) | -1.286 | -1.801 to -0.641 | -3.074 to 1.077 | 0.217 | 0.142 to 0.345 | 0.044 to 0.746 |
|  |  |  | St Georges | 54635 | 1336 (2.4) | -1.992 | -2.562 to -1.437 | -4.741 to 1.303 | 0.120 | 0.072 to 0.192 | 0.009 to 0.786 |
|  |  |  | AMND | 136635 | 3733 (2.7) | -2.238 | -2.791 to -1.652 | -4.987 to 1.023 | 0.096 | 0.058 to 0.161 | 0.007 to 0.735 |
|  |  |  | POP | 4212 | 263 (6.2) | -1.367 | -1.723 to -1.011 | -3.233 to 0.874 | 0.203 | 0.151 to 0.267 | 0.038 to 0.705 |
| 20 | Plasencia 2007c | C | SCOPE | 658 | 26 (4.0) | -4.346 | -4.586 to -4.001 | -5.081 to -1.134 | 0.013 | 0.010 to 0.018 | 0.006 to 0.243 |
|  |  |  | Allen 2017 | 1045 | 13 (1.2) | -4.702 | -5.848 to -4.318 | -6.809 to -1.020 | 0.009 | 0.003 to 0.013 | 0.001 to 0.265 |
|  |  |  | UPBEAT | 1554 | 51 (3.3) | -3.636 | -4.715 to -3.103 | -5.514 to 0.141 | 0.026 | 0.009 to 0.043 | 0.004 to 0.535 |
| 21 | Poon 2010b | C | SCOPE | 658 | 26 (4.0) | -4.460 | -4.746 to -3.925 | -5.459 to -1.342 | 0.011 | 0.009 to 0.019 | 0.004 to 0.207 |
|  |  |  | Allen 2017 | 1045 | 13 (1.2) | -4.542 | -5.007 to -3.860 | -6.748 to -1.142 | 0.011 | 0.007 to 0.021 | 0.001 to 0.242 |
|  |  |  | UPBEAT | 1554 | 51 (3.3) | -3.656 | -4.321 to -2.968 | -5.612 to 0.285 | 0.025 | 0.013 to 0.049 | 0.004 to 0.571 |
| 22 | Scazzocchio 2013b | C | SCOPE | 658 | 26 (4.0) | -3.894 | -4.111 to -3.641 | -4.598 to -1.482 | 0.020 | 0.016 to 0.026 | 0.010 to 0.185 |
| 23 | Poon 2009b | C+B | Allen 2017 | 1045 | 13 (1.2) | -4.505 | -5.108 to -3.883 | -7.179 to -0.850 | 0.011 | 0.006 to 0.020 | 0.001 to 0.305 |
| **Trimester 2 late-onset pre-eclampsia models** | | | |  |  |  |  |  |  |  |  |
| 24 | Yu 2005c | C+U | POP | 4212 | 263 (6.2) | -4.488 | -4.789 to -3.967 | -6.145 to 56.090 | 0.011 | 0.008 to 0.019 | 0.002 to 1.000 |

* For imputed data, estimates are the average across imputations (pooled using Rubin’s rules).

C = Clinical characteristics, C+B = Clinical and biochemical markers, C+U = Clinical characteristics and ultrasound markers

**Table S9: Predictive performance statistics for models in the individual IPPIC-UK cohorts.**

| **Model no.** | **First author (year)** | **Type of predictors** | **Validation cohort** | **N** | **Events***  **(%)** | **Performance statistic (95% CI)** | | |
| --- | --- | --- | --- | --- | --- | --- | --- | --- |
|  |  |  |  |  |  | **C-statistic** | **Calibration slope** | **Calibration-in-the-large** |
| **Trimester 1 any-onset pre-eclampsia models** | | | |  |  |  |  |  |
| 1 | Plasencia 2007a | C | SCOPE | 658 | 33 (5.0) | 0.636 (0.532 to 0.729) | 0.521 (0.025 to 1.017) | 0.865 (0.502 to 1.228) |
|  |  |  | Allen 2017 | 1045 | 14 (1.3) | 0.782 (0.638 to 0.880) | 1.110 (0.600 to 1.619) | -0.117 (-0.651 to 0.417) |
|  |  |  | UPBEAT | 1554 | 56 (3.6) | 0.692 (0.617 to 0.758) | 0.580 (0.346 to 0.813) | -0.326 (-0.610 to -0.042) |
| 2 | Poon 2008 | C | SCOPE | 658 | 33 (5.0) | 0.637 (0.532 to 0.730) | 0.546 (0.051 to 1.040) | 0.731 (0.368 to 1.093) |
|  |  |  | Allen 2017 | 1045 | 14 (1.3) | 0.782 (0.638 to 0.880) | 1.155 (0.622 to 1.689) | -0.209 (-0.743 to 0.324) |
|  |  |  | UPBEAT | 1554 | 56 (3.6) | 0.694 (0.620 to 0.759) | 0.597 (0.357 to 0.837) | -0.518 (-0.801 to -0.234) |
| 3 | Wright 2015a | C | SCOPE | 658 | 33 (5.0) | 0.647 (0.552 to 0.732) | 0.638 (0.097 to 1.179) | 1.880 (1.519 to 2.240) |
|  |  |  | Allen 2017 | 584 | 8 (1.4) | 0.680 (0.453 to 0.845) | 0.840 (-0.100 to 1.781) | 0.367 (-0.334 to 1.069) |
|  |  |  | UPBEAT | 674 | 36 (5.3) | 0.592 (0.497 to 0.681) | 0.560 (-0.067 to 1.187) | 0.544 (0.200 to 0.888) |
| 4 | Baschat 2014a | C+B | Allen 2017 | 1045 | 14 (1.3) | 0.758 (0.620 to 0.857) | 1.246 (0.667 to 1.824) | -1.543 (-2.074 to -1.011) |
|  |  |  | POP | 4212 | 273 (6.5) | 0.704 (0.670 to 0.737) | 1.237 (1.034 to 1.439) | 0.658 (0.533 to 0.782) |
| 5 | Goetzinger 2010 | C+B | Allen 2017 | 1045 | 14 (1.3) | 0.670 (0.502 to 0.804) | 0.910 (0.092 to 1.729) | -1.691 (-2.221 to -1.162) |
|  |  |  | UPBEAT | 1554 | 56 (3.6) | 0.521 (0.451 to 0.590) | 0.409 (-0.567 to 1.386) | -1.206 (-1.480 to -0.933) |
|  |  |  | POP | 4212 | 273 (6.5) | 0.764 (0.730 to 0.795) | 1.706 (1.499 to 1.913) | -0.070 (-0.195 to 0.054) |
| 6 | Odibo 2011a | C+B | Allen 2017 | 1045 | 14 (1.3) | 0.660 (0.491 to 0.796) | 0.847 (-0.022 to 1.716) | -1.512 (-2.041 to -0.982) |
|  |  |  | St Georges | 54635 | 1487 (2.7) | 0.672 (0.654 to 0.690) | 0.962 (0.885 to 1.039) | -0.897 (-0.950 to -0.845) |
|  |  |  | POP | 4212 | 273 (6.5) | 0.779 (0.744 to 0.812) | 1.490 (1.329 to 1.651) | -0.033 (-0.159 to 0.094) |
| 7 | Odibo 2011b | C+U | Velauthar 2012 | 1145 | 28 (2.4) | 0.526 (0.390 to 0.658) | 0.277 (-0.642 to 1.195) | -0.520 (-0.907 to -0.134) |
| **Trimester 2 any-onset pre-eclampsia models** | | | |  |  |  |  |  |
| 8 | Yu 2005a | C+U | POP | 4212 | 273 (6.5) | 0.610 (0.574 to 0.645) | 0.075 (0.007 to 0.144) | NE |
| **Trimester 1 early-onset pre-eclampsia models** | | | |  |  |  |  |  |
| 9 | Baschat 2014b | C | SCOPE | 658 | 6 (0.9) | 0.513 (0.291 to 0.73) | 0.212 (-8.468 to 8.892) | 0.399 (-0.407 to 1.205) |
|  |  |  | ALSPAC | 14344 | 40 (0.3) | 0.706 (0.600 to 0.794) | 2.070 (1.234 to 2.905) | -1.029 (-1.352 to -0.705) |
|  |  |  | Poston 2006 | 2422 | 144 (6.0) | 0.672 (0.626 to 0.716) | 1.281 (0.898 to 1.664) | 1.797 (1.628 to 1.967) |
|  |  |  | Velauthar 2012 | 1145 | 4 (0.3) | 0.698 (0.434 to 0.876) | 1.639 (-4.040 to 7.319) | -0.755 (-1.875 to 0.365) |
|  |  |  | POP | 4212 | 10 (0.2) | 0.739 (0.494 to 0.891) | 3.403 (2.017 to 4.789) | -1.054 (-1.674 to -0.433) |
| 10 | Crovetto 2015a | C | SCOPE | 658 | 6 (0.9) | 0.464 (0.428 to 0.500) | NE | 1.039 (0.218 to 1.861) |
|  |  |  | UPBEAT | 1554 | 6 (0.4) | 0.596 (0.278 to 0.850) | 0.264 (-0.216 to 0.743) | -2.484 (-3.401 to -1.567) |
|  |  |  | POP | 4212 | 10 (0.2) | 0.721 (0.532 to 0.855) | 0.996 (0.596 to 1.395) | -0.317 (-0.943 to 0.309) |
| 11 | Kuc 2013a | C | SCOPE | 658 | 6 (0.9) | 0.762 (0.520 to 0.905) | 0.682 (0.050 to 1.314) | -3.709 (-4.530 to -2.889) |
|  |  |  | ALSPAC | 14344 | 40 (0.3) | 0.664 (0.484 to 0.812) | 0.442 (-0.025 to 0.909) | -3.458 (-3.786 to -3.130) |
|  |  |  | UPBEAT | 1554 | 6 (0.4) | 0.519 (0.323 to 0.710) | 0.010 (-0.705 to 0.726) | -5.978 (-6.861 to -5.094) |
|  |  |  | St Georges | 54635 | 151 (0.3) | 0.635 (0.587 to 0.679) | 0.343 (0.227 to 0.460) | -4.510 (-4.674 to -4.347) |
|  |  |  | AMND | 136635 | 1237 (0.9) | 0.681 (0.665 to 0.696) | 0.470 (0.430 to 0.511) | -3.387 (-3.445 to -3.330) |
|  |  |  | POP | 4212 | 10 (0.2) | 0.656 (0.518 to 0.772) | 0.450 (-0.069 to 0.969) | -5.191 (-5.815 to -4.567) |
| 12 | Plasencia 2007b | C | SCOPE | 658 | 6 (0.9) | 0.465 (0.429 to 0.501) | NE | 1.500 (0.691 to 2.309) |
|  |  |  | Chappell 1999 | 316 | 7 (2.1) | 0.706 (0.463 to 0.872) | 0.510 (0.013 to 1.007) | 0.462 (-0.402 to 1.327) |
|  |  |  | UPBEAT | 1554 | 6 (0.4) | 0.667 (0.391 to 0.863) | 0.517 (-0.135 to 1.170) | -0.478 (-1.367 to 0.411) |
|  |  |  | POP | 4212 | 10 (0.2) | 0.497 (0.496 to 0.498) | NE | 0.365 (-0.256 to 0.985) |
| 13 | Poon 2010a | C | SCOPE | 658 | 6 (0.9) | 0.524 (0.355 to 0.687) | 0.236 (-1.154 to 1.627) | 0.648 (-0.163 to 1.458) |
|  |  |  | UPBEAT | 1554 | 6 (0.4) | 0.745 (0.512 to 0.892) | 0.846 (0.004 to 1.688) | -1.667 (-2.548 to -0.787) |
|  |  |  | POP | 4212 | 10 (0.2) | 0.687 (0.455 to 0.852) | 1.176 (0.617 to 1.734) | -2.228 (-2.849 to -1.606) |
| 14 | Scazzocchio 2013a | C | SCOPE | 658 | 6 (0.9) | 0.728 (0.448 to 0.898) | 1.131 (-0.130 to 2.392) | 0.788 (-0.020 to 1.596) |
|  |  |  | UPBEAT | 1554 | 6 (0.4) | 0.612 (0.333 to 0.834) | 0.586 (-0.376 to 1.548) | -1.434 (-2.316 to -0.551) |
|  |  |  | POP | 4212 | 10 (0.2) | 0.844 (0.640 to 0.943) | 0.746 (0.449 to 1.042) | -1.439 (-2.092 to -0.787) |
| 15 | Wright 2015b | C | SCOPE | 658 | 6 (0.9) | 0.763 (0.614 to 0.867) | 0.907 (-0.027 to 1.842) | 1.396 (0.585 to 2.207) |
|  |  |  | UPBEAT | 674 | 6 (0.4) | 0.591 (0.207 to 0.890) | 0.958 (-0.727 to 2.642) | -0.906 (-2.048 to 0.236) |
| 16 | Poon 2009a | C+B | POP | 4212 | 10 (0.2) | 0.741 (0.507 to 0.888) | 0.452 (0.210 to 0.693) | -2.671 (-3.35 to -1.991) |
| **Trimester 2 early-onset pre-eclampsia models** | | | |  |  |  |  |  |
| 17 | Yu 2005b | C+U | POP | 4212 | 10 (0.2) | 0.908 (0.826 to 0.954) | 0.557 (0.293 to 0.821) | 2.473 (1.716 to 3.229) |
| **Trimester 1 late-onset pre-eclampsia models** | | | |  |  |  |  |  |
| 18 | Crovetto 2015b | C | SCOPE | 658 | 26 (4.0) | 0.569 (0.448 to 0.683) | 0.379 (-0.231 to 0.988) | 1.074 (0.675 to 1.472) |
|  |  |  | Allen 2017 | 1045 | 13 (1.2) | 0.544 (0.352 to 0.723) | 0.516 (0.108 to 0.924) | -1.205 (-1.779 to -0.630) |
|  |  |  | Chappell 1999 | 316 | 32 (10.0) | 0.696 (0.586 to 0.788) | 0.345 (0.152 to 0.538) | 0.037 (-0.451 to 0.525) |
|  |  |  | UPBEAT | 1554 | 51 (3.3) | 0.504 (0.412 to 0.596) | 0.210 (-0.035 to 0.456) | -1.502 (-1.812 to -1.192) |
|  |  |  | POP | 4212 | 263 (6.2) | 0.781 (0.745 to 0.813) | 1.248 (1.120 to 1.376) | 1.309 (1.177 to 1.441) |
| 19 | Kuc 2013b | C | SCOPE | 658 | 26 (4.0) | 0.544 (0.421 to 0.663) | 0.224 (-0.445 to 0.893) | -1.867 (-2.262 to -1.471) |
|  |  |  | Allen 2017 | 1045 | 13 (1.2) | 0.583 (0.425 to 0.726) | 0.340 (-0.356 to 1.036) | -2.570 (-3.119 to -2.020) |
|  |  |  | ALSPAC | 14344 | 266 (1.9) | 0.657 (0.616 to 0.696) | 0.761 (0.550 to 0.973) | -1.574 (-1.699 to -1.448) |
|  |  |  | EMPOWAR | 449 | 28 (6.3) | 0.337 (0.162 to 0.558) | -0.755 (-1.808 to 0.299) | -2.127 (-2.795 to -1.459) |
|  |  |  | UPBEAT | 1554 | 51 (3.3) | 0.592 (0.512 to 0.668) | 0.390 (0.019 to 0.762) | -2.435 (-2.724 to -2.147) |
|  |  |  | St Georges | 54635 | 1336 (2.4) | 0.636 (0.621 to 0.651) | 0.632 (0.560 to 0.704) | -1.970 (-2.025 to -1.915) |
|  |  |  | AMND | 136635 | 3733 (2.7) | 0.844 (0.640 to 0.943) | 0.746 (0.449 to 1.042) | -1.439 (-2.092 to -0.787) |
|  |  |  | POP | 4212 | 263 (6.2) | 0.599 (0.561 to 0.636) | 0.673 (0.452 to 0.894) | -1.487 (-1.613 to -1.361) |
| 20 | Plasencia 2007c | C | SCOPE | 658 | 26 (4.0) | 0.627 (0.509 to 0.732) | 0.523 (-0.003 to 1.049) | 0.778 (0.377 to 1.178) |
|  |  |  | Allen 2017 | 1045 | 13 (1.2) | 0.751 (0.600 to 0.858) | 1.008 (0.459 to 1.556) | 0.043 (-0.510 to 0.596) |
|  |  |  | UPBEAT | 1554 | 51 (3.3) | 0.673 (0.596 to 0.742) | 0.523 (0.264 to 0.782) | -0.204 (-0.498 to 0.090) |
| 21 | Poon 2010b | C | SCOPE | 658 | 26 (4.0) | 0.570 (0.405 to 0.682) | 0.369 (-0.199 to 0.937) | 0.909 (0.511 to 1.308) |
|  |  |  | Allen 2017 | 1045 | 13 (1.2) | 0.716 (0.556 to 0.836) | 0.913 (0.312 to 1.514) | -0.289 (-0.841 to 0.262) |
|  |  |  | UPBEAT | 1554 | 51 (3.3) | 0.666 (0.591 to 0.734) | 0.541 (0.280 to 0.802) | -0.271 (-0.566 to 0.025) |
| 22 | Scazzocchio 2013b | C | SCOPE | 658 | 26 (4.0) | 0.597 (0.478 to 0.705) | 0.562 (-0.168 to 1.291) | 0.524 (0.128 to 0.920) |
| 23 | Poon 2009b | C+B | Allen 2017 | 1045 | 13 (1.2) | 0.684 (0.550 to 0.792) | 0.799 (0.257 to 1.341) | -0.349 (-0.902 to 0.205) |
| **Trimester 2 late-onset pre-eclampsia models** | | | |  |  |  |  |  |
| 24 | Yu 2005c | C+U | POP | 4212 | 263 (6.2) | 0.607 (0.570 to 0.642) | 0.077 (0.005 to 0.148) | NE |

* For datasets with imputed outcomes, estimates are averages across 100 imputations (pooled using Rubin’s rules).

C = Clinical characteristics, C+B = Clinical and biochemical markers, C+U = Clinical characteristics and ultrasound markers

NE = Not estimable due to perfect prediction (same predicted probability for all individuals that had the event).

Table S10: Predictive performance statistics for models in nulliparous women in all cohorts and in the POP cohort.

| **Model no.** | **Author (year)** | **Type of predictor** | **Summary estimates for nulliparous women only (95% CI),** | | | | | | **POP (4212 women)** | | |
| --- | --- | --- | --- | --- | --- | --- | --- | --- | --- | --- | --- |
|  |  |  | **No of validation cohorts** | **No of women** | **Total events** | **C-statistic** | **Calibration slope** | **Calibration-in-the-large** | **C-statistic**  **(95% CI)** | **Calibration slope**  **(95% CI)** | **Calibration in the large**  **(95% CI)** |
| **Any-onset pre-eclampsia models** | | | | | | | | | | | |
| 1 | Plasencia 2007a | Clinical | 3 | 1919 | 76 | 0.62  (0.47, 0.75) | 0.50  (-0.17, 1.16) | 0.11  (-1.63, 1.84) |  |  |  |
| 2 | Poon 2008 |  | 2 | 1919 | 76 | 0.62  (0.47, 0.75) | 0.52  (-0.16, 1.19) | -0.04  (-1.79, 1.71) |  |  |  |
| 3 | Wright 2015a |  | 3 | 1916 | 76 | 0.62  (0.48, 0.75) | 0.64  (-0.18, 1.47) | 0.95  (-1.13, 3.03) |  |  |  |
| 4 | Baschat 2014a | Clinical and biochemical | 2 | 4796 | 281 | 0.71  (0.46, 0.87) | 1.22  (-0.05, 2.50) | -0.44  (-14.69, 13.81) | 0.71  (0.67, 0.74) | 1.24  (1.03, 1.44) | 0.66  (0.53, 0.78) |
| 5 | Goetzinger 2010 |  | 3 | 5473 | 317 | 0.66  (0.30, 0.90) | 1.25  (-0.41, 2.92) | -0.75  (-2.60, 1.10) | 0.76  (0.73, 0.80) | 1.71  (1.50, 1.91) | -0.07  (-0.20, 0.05) |
| 6 | Odibo 2011a |  | 3 | 34174 | 1238 | 0.71  (0.46, 0.88) | 1.15  (0.12, 2.18) | -0.63  (-2.29, 1.04) | 0.78  (0.74, 0.81) | 1.49  (1.33, 1.65) | -0.03  (-0.16, 0.09) |
| 7 | Odibo 2011b | Clinical and ultrasound | 1 | 598 | 21 | 0.51  (0.35, 0.67) | 0.21  (-0.95, 1.36) | -0.03  (-0.47, 0.42) |  |  |  |
| 8 | Yu 2005a |  | 1 | 4212 | 273 | 0.61  (0.57, 0.65) | 0.08  (0.01, 0.14) | NE | 0.61  (0.57, 0.65) | 0.08  (0.01, 0.14) | NE |
| **Early-onset pre-eclampsia models** | | | | | | | | | | | |
| 9 | Baschat 2014b | Clinical | 5 | 11959 | 47 | 0.67  (0.51, 0.80) | 2.68  (1.09, 4.26) | -0.41  (-1.38, 0.57) | 0.74  (0.50, 0.89) | 3.40  (2.02, 4.79) | -1.05  (-1.67, -0.43) |
| 10 | Crovetto 2015a |  | 3 | 5547 | 19 | 0.57  (0.21, 0.87) | 0.84  (-2.71, 4.38) | 0.21  (-1.71, 2.12) | 0.72  (0.53, 0.86) | 1.00  (0.60, 1.40) | -0.32  (-0.94, 0.31) |
| 11 | Kuc 2013a |  | 6 | 106623 | 968 | 0.63  (0.57, 0.68) | 0.4  (0.27, 0.53) | -4.46  (-5.59, -3.33) | 0.66  (0.52, 0.77) | 0.45  (-0.07, 0.97) | -5.19  (-5.82, -4.57) |
| 12 | Plasencia 2007b |  | 4 | 5749 | 22 | 0.49  (0.44, 0.53) | 0.71  (-5.88, 7.30) | 0.94  (-0.14, 2.03) | 0.50  (0.50, 0.50) | NE | 0.37  (-0.26, 0.99 |
| 13 | Poon 2010a |  | 3 | 5547 | 19 | 0.58  (0.31, 0.81) | 0.98  (-0.28, 2.24) | -1.05  (-4.86, 2.76) | 0.69  (0.46, 0.85) | 1.18  (0.62, 1.73) | -2.23  (-2.85, -1.61) |
| 14 | Scazzocchio 2013a |  | 3 | 5547 | 19 | 0.75  (0.44, 0.92) | 0.76  (0.14, 1.39) | -0.51  (-3.48, 2.46) | 0.84  (0.64 to 0.94) | 0.75  (0.45, 1.04) | -1.44  (-2.09, -0.79) |
| 15 | Wright 2015b |  | 2 | 1332 | 9 | 0.74  (0.04, 1.00) | 0.92  (-4.39, 6.22) | 0.28  (-14.34, 14.9) |  |  |  |
| 16 | Poon 2009a | Clinical and biochemical | 1 | 4212 | 10 | 0.74  (0.51, 0.89) | 0.45  (0.21 to 0.69) | -2.67  (-3.35 to -1.99) | 0.74  (0.51, 0.89) | 0.45  (0.21 to 0.69) | -2.67  (-3.35 to -1.99) |
| 17 | Yu 2005b | Clinical and ultrasound | 1 | 4212 | 10 | 0.91  (0.83, 0.95) | 0.56  (0.29, 0.82) | 2.47  (1.716, 3.23) | 0.91  (0.83, 0.95) | 0.56  (0.29, 0.82) | 2.47  (1.716, 3.23) |
| **Late-onset pre-eclampsia models** | | | | | | | | | | | |
| 18 | Crovetto 2015b | Clinical | 5 | 6333 | 345 | 0.64  (0.47, 0.78) | 0.68  (0.012, 1.33) | 0.78  (-0.16, 1.73) | 0.78  (0.75, 0.81) | 1.25  (1.12, 1.38) | 1.31  (1.18, 1.44) |
| 19 | Kuc 2013b |  | 8 | 107498 | 4195 | 0.58  (0.54, 0.61) | 0.49  (0.27, 0.71 | -2.00  (-2.42, -1.58) | 0.60  (0.56, 0.64) | 0.67  (0.45, 0.89) | -1.49  (-1.61, -1.36) |
| 20 | Plasencia 2007c |  | 3 | 1919 | 67 | 0.61  (0.45, 0.75) | 0.47  (-0.26, 1.2) | 0.11  (-1.41, 1.64) |  |  |  |
| 21 | Poon 2010b |  | 3 | 1919 | 67 | 0.56  (0.39, 0.71) | 0.29  (-0.48, 1.06) | 0.13  (-1.64, 1.91) |  |  |  |
| 22 | Scazzocchio 2013b |  | 1 | 658 | 26 | 0.60  (0.48, 0.71) | 0.56  (-0.17, 1.29) | 0.52  (0.13, 0.92) |  |  |  |
| 23 | Poon 2009b | Clinical and biochemical | 1 | 584 | 8 | 0.63  (0.43, 0.79) | 0.83  (-0.00, 1.66) | -0.46  (-1.17, 0.24) |  |  |  |
| 24 | Yu 2005c | Clinical and ultrasound | 1 | 4212 | 263 | 0.61  (0.57, 0.64) | 0.08  (0.01, 0.15) | NE | 0.61  (0.57, 0.64) | 0.08  (0.01, 0.15) | NE |

* Estimates are averages across imputations (pooled using Rubin’s rules).

NE = Not estimable due to perfect prediction (same predicted probability for all individuals that had the event).

Figure S1: Decision curves for early pre-eclampsia models in SCOPE, UPBEAT and POP.


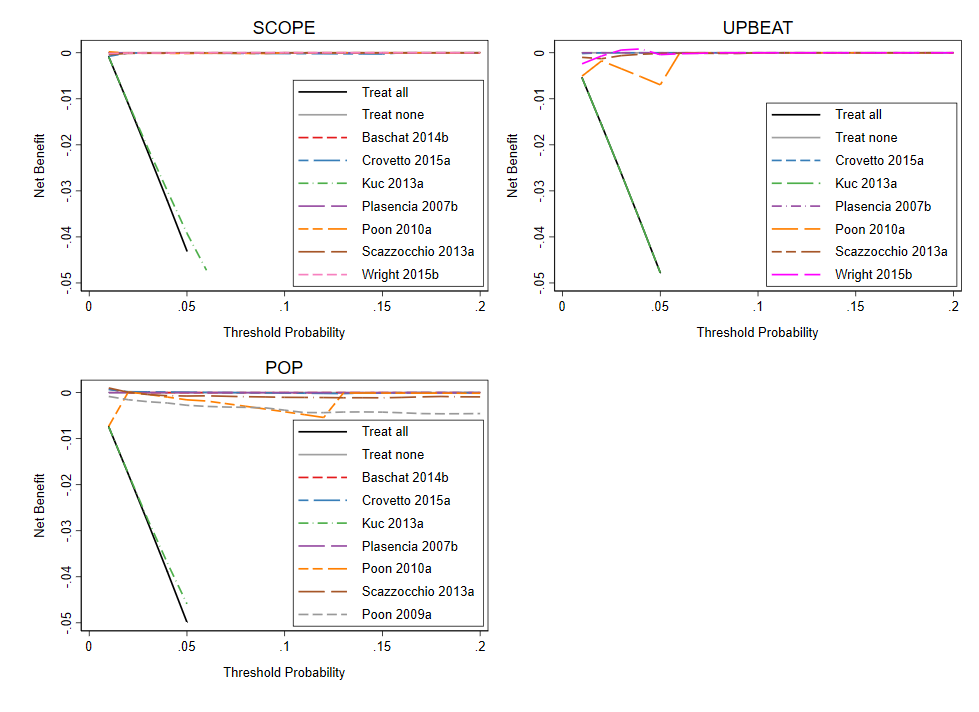


Figure S2: Decision curves for late pre-eclampsia models in SCOPE, Allen 2017, UPBEAT and POP.


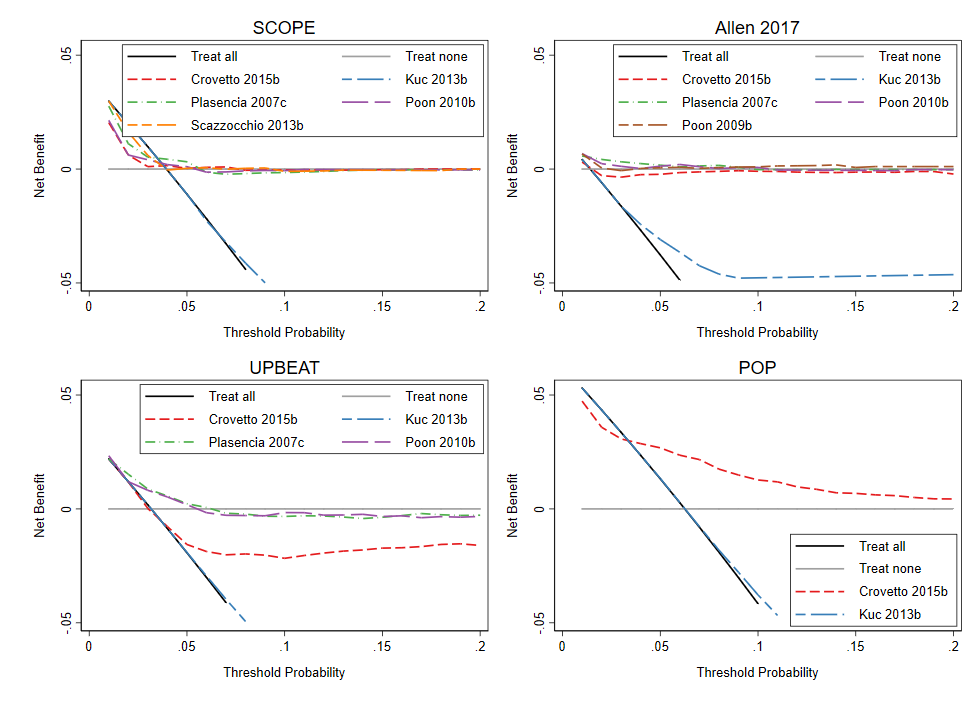

Supplement: Supplementary file 1 — Additional file 1: Supplementary methods: Additional details for handling missing data and evaluating predictive performance of models. Table S1: Search strategy for pre-eclampsia prediction models. Table S2: Predictors evaluated in the models externally validated in the IPPIC-UK cohorts. Table S3: Prediction models and equations identified from the literature search. Table S4: Study level characteristics of IPPIC-UK cohorts. Table S5: Patient characteristics of IPPIC-UK cohorts. Table S6: Number and proportion missing for each predictor in each cohort used for external validation. Table S7: Risk of bias assessment of the IPPIC-UK cohorts using the PROBAST tool. Table S8: Summary of linear predictor values and predicted probabilities for each model in each cohort. Table S9: Predictive performance statistics for models in the individual IPPIC-UK cohorts. Table S10: Predictive performance statistics for models in nulliparous women in all cohorts and in the POP cohort. Fig. S1: Decision curves for early pre-eclampsia models in SCOPE, UPBEAT and POP. Fig. S2: Decision curves for late pre-eclampsia models in SCOPE, Allen 2017, UPBEAT and POP. [file 12916_2020_1766_MOESM1_ESM.docx]
